# Supplementary material for: Synergistic correlated states and nontrivial topology in coupled graphene-insulator heterostructures
Source: Nat Commun. 2023 Sep 9;14:5550. doi: 10.1038/s41467-023-41293-8 (PMC10492827; doi:10.1038/s41467-023-41293-8)
Supplement: Supplementary file 1 — Supplementary Information [file 41467_2023_41293_MOESM1_ESM.pdf]

Supplementary Information for "Synergistic correlated states and nontrivial topology in coupled graphene-insulator heterostructures"

Xin Lu,[1] Shihao Zhang,[1] Yaning Wang,[2] Xiang Gao,[3,4] Kaining Yang,[3,4] Zhongqing Guo,[1] Yuchen Gao,[5,6] Yu Ye,[5,6] Zheng Vitto Han,[3,4] and Jianpeng Liu[1,7,*]

[1]*School of Physical Science and Technology, ShanghaiTech University, Shanghai 201210, China*
[2]*Shenyang National Laboratory for Materials Science, Institute of Metal Research, Chinese Academy of Sciences, Shenyang, China*
[3]*State Key Laboratory of Quantum Optics and Quantum Optics Devices, Institute of Opto-Electronics, Shanxi University, 030006 Taiyuan, China*
[4]*Collaborative Innovation Center of Extreme Optics, Shanxi University, 030006 Taiyuan, China*
[5]*Collaborative Innovation Center of Quantum Matter, Beijing 100871, China*
[6]*State Key Lab for Mesoscopic Physics and Frontiers Science Center for Nano-Optoelectronics, School of Physics, Peking University, Beijing 100871, China*
[7]*ShanghaiTech Laboratory for Topological Physics, ShanghaiTech University, Shanghai 201210, China*

CONTENTS

Supplementary Note 1: Non-interacting Hamiltonian for a graphene-insulator heterostructure 2

Supplementary Note 2: Topological Properties of the non-interacting Hamiltonian 5

Supplementary Note 3: Renormalization group derivations 8
- Electron-electron interaction in a lower energy window 9
- Evaluation of the correction to the non-interacting Hamiltonian from the fast modes 10
- Renormalization group flow equations 11

Supplementary Note 4: Hartree-Fock approximations to electron-electron interactions 12

Supplementary Note 5: Results of Hartree-Fock calculations 15

Supplementary Note 6: General coupled bilayer system in graphene-insulator heterostructures 17
- Hamiltonians 17
- Hartree-Fock calculations on the coupled bilayer system 20

Supplementary Note 7: Details of DFT calculations for the substrate materials 23
- Lattice structures, deformation potentials, and band structures of candidate substrate materials 23
- Band structure of graphene-CrOCl heterostructure 24
- Electric-field tunable band structures of bilayer CrOCl 26
- Transition metal dichalcogenides as substrates for graphene 26

Supplementary Note 8: Experimental measurements of the gaps in graphene-CrOCl heterostructure 28
- Quality of sample, device configuration and measurement setup 28
- Measurement of the thermal gap 30

Supplementary references 32

* liujp@shanghaitech.edu.cn

SUPPLEMENTARY NOTE 1: NON-INTERACTING HAMILTONIAN FOR A GRAPHENE-INSULATOR HETEROSTRUCTURE

The Hamiltonian for a graphene-insulator heterostructure can be always divided into three parts: graphene part H_G, the insulating substrate part H_S and the coupling between them $H_{G-\mathrm{S}}$. The graphene part can be suitably described by a tight-binding model since we focus on the low-energy physics. As we have explained in the main text, with slight carrier doping band edge, the insulator substrate is supposed to form a long-wavelength charge order on the interface near graphene sheet thanks to Coulomb interactions between electrons occupying the band edge of the insulating substrate (transferred from graphene layer). The insulator substrate part is then modeled by a 2D Hamiltonian for electrons hopping on a 2D superlattice which forms a Wigner-crystal-like or long-wavelength charge ordered insulator state at some proper filling. The geometry of the superlattice is determined by the long-wavelength order at the interface. Explicitly, the graphene part H_G and the insulator substrate part H_S can be generally written as

$$\hat{H}_G = \sum_{\mathbf{k},\sigma,\alpha,\alpha'} \gamma_{\alpha,\alpha'}(\mathbf{k})\hat{c}^{\dagger}_{\sigma\alpha}(\mathbf{k})\hat{c}_{\sigma\alpha'}(\mathbf{k}) \tag{S1}$$

$$\hat{H}_S = \sum_{\widetilde{\mathbf{k}},\sigma} \eta(\widetilde{\mathbf{k}})\hat{d}^{\dagger}_{\sigma}(\widetilde{\mathbf{k}})\hat{d}_{\sigma}(\widetilde{\mathbf{k}}) \tag{S2}$$

where $\hat{c}^{(\dagger)}_{\sigma\alpha}(\mathbf{k})$ and $\hat{d}^{(\dagger)}_{\sigma}(\widetilde{\mathbf{k}})$ are fermionic annihilation (creation) operators for electrons in graphene and the insulator substrate, respectively. In the lower index of these operators, α is the sublattice index for the bipartite lattice of graphene and σ is the spin degree of freedom of electrons. To emphasize the fact that graphene and the insulator substrate have different lattices and thus different Brillouin zone, we denote $\mathbf{k}$ and $\widetilde{\mathbf{k}}$ as the wavevectors in the Brillouin zone of graphene and that of the long-wavelength superlattice in the substrate, respectively. In our calculations, the lattice for H_S is set to be rectangular or triangular. The lattice symmetry turns out to make no qualitative differences.

Since electrons have negligible probability to hop between graphene and the insulator substrate due to rather large distance d between two sheets in the z-direction ($d \sim 7\,\text{Å}$ from DFT calculations in CrOCl-graphene heterostructure), we suppose that electrons from two sheets are coupled only via long-ranged Coulomb interactions. Unlike H_G and H_S, such long-ranged Coulomb interactions are more easily written in real space. In terms of field operators $\hat{\psi}(\mathbf{r})$, the inter-sheet coupling reads

$$\hat{H}_{G-\mathrm{S}} = \int d^2\mathbf{r}d^2\mathbf{r}' \sum_{\sigma,\sigma'} \hat{\psi}^{\dagger}_{c,\sigma}(\mathbf{r})\hat{\psi}^{\dagger}_{d,\sigma'}(\mathbf{r}')V(|\mathbf{r}-\mathbf{r}'+d\hat{\mathbf{z}}|)\hat{\psi}_{d,\sigma'}(\mathbf{r}')\hat{\psi}_{c,\sigma}(\mathbf{r}) \tag{S3}$$

where $V(|\mathbf{r}-\mathbf{r}'+d\hat{\mathbf{z}}|)$ is the 3D long-ranged Coulomb potential $e^2/4\pi\epsilon_0\epsilon_r r$ and electrons in graphene and the insulating substrate are described by the field operators with lower index c and d, respectively. Here ϵ_0 is the vacuum permittivity and ϵ_r is the dimensionless relative dielectric constant of the insulating substrate. In the spirit of tight-binding formalism, we write the field operators in terms of Wannier functions

$$\hat{\psi}^{\dagger}_{c,\sigma}(\mathbf{r}) = \sum_{i,\alpha} \phi^{*}_{\alpha}(\mathbf{r}-\mathbf{a}_i-\boldsymbol{\tau}_{\alpha})\chi^{\dagger}_{\sigma}\hat{c}^{\dagger}_{i,\sigma\alpha} \tag{S4}$$

$$\hat{\psi}^{\dagger}_{d,\sigma}(\mathbf{r}) = \sum_{i,\alpha} \widetilde{\phi}^{*}(\mathbf{r}-\mathbf{R}_i)\chi^{\dagger}_{\sigma}\hat{d}^{\dagger}_{i,\sigma} \tag{S5}$$

where ϕ_{α} and $\widetilde{\phi}$ are Wannier functions localized on the graphene and the insulator substrate Bravais lattice sites, which are described by $\mathbf{a}_i$ and $\mathbf{R}_i$, respectively. Here α refers to the sublattice index in graphene and $\boldsymbol{\tau}_{\alpha}$ is the vector denoting the position of the αth sublattice inside the unit-cell. The spin degrees of freedom is included by the index σ and also explicitly by spinor χ_{σ}. It is worthwhile to note that here the Bravais lattice sites and the corresponding Wannier functions for the substrate refer to those of the spontaneously generated charge ordered superlattice, not the atomic lattices of the substrate. A more fundamental treatment based on the atomic lattice sites of the substrate lattice will be discussed in Supplementary Note 6.

After the transformations above, the Hamiltonian $H_{G-\mathrm{S}}$ in the Wannier basis reads

$$\hat{H}_{G-\mathrm{S}} = \sum_{\substack{\sigma,\sigma' \\ \alpha,\alpha'}} \sum_{\substack{i,i' \\ j,j'}} U^{\sigma\sigma'}_{i\alpha j,i'\alpha' j'}\hat{c}^{\dagger}_{i,\sigma\alpha}\hat{d}^{\dagger}_{j,\sigma'}\hat{d}_{j',\sigma'}\hat{c}_{i',\sigma\alpha'} \tag{S6}$$

with

$$U^{\sigma\sigma'}_{i\alpha j,i'\alpha' j'} = \int d^2\mathbf{r} d^2\mathbf{r}' \phi^*_\alpha(\mathbf{r}-\mathbf{a}_i-\boldsymbol{\tau}_\alpha)\widetilde{\phi}^*(\mathbf{r}-\mathbf{R}_j)V(|\mathbf{r}-\mathbf{r}'+d\hat{\mathbf{z}}|)\widetilde{\phi}(\mathbf{r}'-\mathbf{R}_{j'})\phi_{\alpha'}(\mathbf{r}-\mathbf{a}_{i'}-\boldsymbol{\tau}_{\alpha'})\chi^\dagger_\sigma\chi^\dagger_{\sigma'}\chi_{\sigma'}\chi_\sigma. \tag{S7}$$

If Wannier functions are so localized such that

$$\phi^*_\alpha(\mathbf{r}-\mathbf{a}_i-\boldsymbol{\tau}_\alpha)\phi_{\alpha'}(\mathbf{r}-\mathbf{a}_{i'}-\boldsymbol{\tau}_{\alpha'}) \approx 0 \qquad \text{if } (i,\alpha)\neq(i',\alpha') \tag{S8}$$

$$\widetilde{\phi}^*(\mathbf{r}-\mathbf{R}_j)\widetilde{\phi}(\mathbf{r}-\mathbf{R}_{j'}) \approx 0 \qquad \text{if } j\neq j' \tag{S9}$$

$$|\phi_\alpha(\mathbf{r}-\mathbf{a}_i-\boldsymbol{\tau}_\alpha)|^2 \approx \delta^{(2)}(\mathbf{r}-\mathbf{a}_i-\boldsymbol{\tau}_\alpha) \tag{S10}$$

$$|\widetilde{\phi}(\mathbf{r}-\mathbf{R}_j)|^2 \approx \delta^{(2)}(\mathbf{r}-\mathbf{R}_j)\ , \tag{S11}$$

with $\delta^{(2)}(\mathbf{r})$ is the 2D Dirac δ-function distribution, we can simplify the previous expression to

$$U^{\sigma\sigma'}_{i\alpha j,i'\alpha' j'} = U_{i\alpha j}\delta_{i,i'}\delta_{\alpha,\alpha'}\delta_{j,j'} \tag{S12}$$

with $\delta_{\mu,\nu}$ is the Kronecker delta and

$$U_{i\alpha j} = V(|\mathbf{a}_i+\boldsymbol{\tau}_\alpha-\mathbf{R}_j+d\hat{\mathbf{z}}|). \tag{S13}$$

Then, we write H_{G-S} in reciprocal space using the following Fourier transformation

$$\hat{c}_{i,\sigma\alpha} = \frac{1}{\sqrt{N_c}}\sum_{\mathbf{k}} e^{i\mathbf{k}\cdot\mathbf{a}_i}\hat{c}_{\sigma\alpha}(\mathbf{k}) \tag{S14}$$

$$\hat{d}_{i,\sigma} = \frac{1}{\sqrt{N_d}}\sum_{\widetilde{\mathbf{k}}} e^{i\widetilde{\mathbf{k}}\cdot\mathbf{R}_i}\hat{d}_\sigma(\widetilde{\mathbf{k}}) \tag{S15}$$

where N_c and N_d are the number of lattice sites for electron in graphene and the insulator substrate, respectively. The Hamiltonian $H_{G-\mathrm{S}}$ in the basis of $\hat{c}_{\sigma\alpha}(\mathbf{k})$ and $\hat{d}_\sigma(\widetilde{\mathbf{k}})$ reads

$$\hat{H}_{G-S} = \frac{1}{N_cN_d}\sum_{\substack{\sigma,\sigma'\\ i,\alpha,j}}\sum_{\substack{\mathbf{k},\mathbf{k}'\\ \widetilde{\mathbf{k}},\widetilde{\mathbf{k}}'}} U_{i\alpha j}\ e^{i(\mathbf{k}'-\mathbf{k})\cdot(\mathbf{a}_i-\mathbf{R}_j)}\ e^{i(\mathbf{k}'-\mathbf{k}+\widetilde{\mathbf{k}}'-\widetilde{\mathbf{k}})\cdot\mathbf{R}_j}\ \hat{c}^\dagger_{\sigma\alpha}(\mathbf{k})\hat{d}^\dagger_{\sigma'}(\widetilde{\mathbf{k}})\hat{d}_{\sigma'}(\widetilde{\mathbf{k}}')\hat{c}_{\sigma\alpha}(\mathbf{k}'). \tag{S16}$$

Now we first define $\widetilde{\mathbf{R}} = \mathbf{a}_i - \mathbf{R}_j$, and let $\mathbf{k}'-\mathbf{k} = \mathbf{q} = \widetilde{\mathbf{q}}+\mathbf{G}$, where $\mathbf{G}$ is a reciprocal vector of the long-wavelength ordered superlattice and $\widetilde{\mathbf{q}}$ is the wavevector within the superlattice Brillouin zone. Then we take use of the identity $\sum_j e^{i(\widetilde{\mathbf{k}}'-\widetilde{\mathbf{k}}+\mathbf{q})\cdot\mathbf{R}_j} = \sum_j e^{i(\widetilde{\mathbf{k}}'-\widetilde{\mathbf{k}}+\widetilde{\mathbf{q}}+\mathbf{G})\cdot\mathbf{R}_j} = N_d\delta_{\widetilde{\mathbf{k}}'-\widetilde{\mathbf{k}},\widetilde{\mathbf{q}}}$, Eq. (S16) can be simplified as

$$\hat{H}_{G-S} = \sum_{\substack{\sigma,\sigma'\\ \alpha}}\sum_{\substack{\mathbf{k},\widetilde{\mathbf{k}}\\ \widetilde{\mathbf{q}},\widetilde{\mathbf{G}}}} \widetilde{V}(\widetilde{\mathbf{q}}+\mathbf{G})\ \hat{c}^\dagger_{\sigma\alpha}(\mathbf{k})\hat{d}^\dagger_{\sigma'}(\widetilde{\mathbf{k}})\hat{d}_{\sigma'}(\widetilde{\mathbf{k}}+\widetilde{\mathbf{q}})\hat{c}_{\sigma\alpha}(\mathbf{k}-\widetilde{\mathbf{q}}-\mathbf{G}) \tag{S17}$$

The coupling $\widetilde{V}(\widetilde{\mathbf{q}}+\mathbf{G})$ reads

$$\begin{aligned}
\widetilde{V}(\widetilde{\mathbf{q}}+\mathbf{G}) &= \frac{1}{N_c}\sum_i V(|\mathbf{a}_i+\boldsymbol{\tau}_\alpha-\mathbf{R}_j+d\hat{\mathbf{z}}|)e^{-i(\widetilde{\mathbf{q}}+\mathbf{G})\cdot(\mathbf{a}_i-\mathbf{R}_j)}\\
&= \frac{1}{N_c}\sum_{\widetilde{\mathbf{R}}} V(|\widetilde{\mathbf{R}}+\boldsymbol{\tau}_\alpha+d\hat{\mathbf{z}}|)e^{-i(\widetilde{\mathbf{q}}+\mathbf{G})\cdot\widetilde{\mathbf{R}}}\\
&= \frac{1}{N_d}\int\frac{d^2r}{\Omega_d}V(|\mathbf{r}+\boldsymbol{\tau}_\alpha+d\hat{\mathbf{z}}|)e^{-i(\widetilde{\mathbf{q}}+\mathbf{G})\cdot\mathbf{r}}\\
&= \frac{e^2}{2\epsilon_0\epsilon_r N_d\Omega_d}\frac{e^{-|\widetilde{\mathbf{q}}+\mathbf{G}|d}}{|\widetilde{\mathbf{q}}+\mathbf{G}|}
\end{aligned} \tag{S18}$$

where Ω_d is the area of the unit-cell of the surface superlattice of the substrate. In the third line of the above derivation, we smear the sum over $\widetilde{\mathbf{R}} = \mathbf{a}_i - \mathbf{R}_j$ by replacing it with an integral over the surface $S = N_d\Omega_d = N_c\Omega_c$ with Ω_c the

area of graphene's unit-cell since we are interested in the physics in the length scale of the superlattice $\{\mathbf{R}_j\}$, which is supposed to much larger than that of graphene. Finally, the last line is the 2D partial Fourier transformation of the 3D Coulomb potential.

Since we focus on the low-energy physics around the Dirac cones of graphene, we can attribute valley index μ to electrons in graphene and neglect intervalley coupling thanks to the exponential decay of $\widetilde{V}(\mathbf{q})$ so that

$$\hat{H}_{G-S} = \sum_{\substack{\sigma,\sigma' \\ \alpha}} \sum_{\substack{\mathbf{k},\widetilde{\mathbf{k}} \\ \widetilde{\mathbf{q}},\mathbf{G}}} \widetilde{V}(\widetilde{\mathbf{q}}+\mathbf{G}) \sum_{\mu} \hat{c}^{\dagger}_{\sigma\mu\alpha}(\mathbf{k}) \hat{d}^{\dagger}_{\sigma'}(\widetilde{\mathbf{k}}) \hat{d}_{\sigma'}(\widetilde{\mathbf{k}}+\widetilde{\mathbf{q}}) \hat{c}_{\sigma\mu\alpha}(\mathbf{k}-\widetilde{\mathbf{q}}-\mathbf{G}). \tag{S19}$$

In the meantime, the Hamiltonian for graphene only H_G [see Eq. (S1)] can be divided into two valley sectors

$$\hat{H}_G = \sum_{\mathbf{k},\sigma,\alpha,\alpha',\mu} \left(\hbar v_F \mathbf{k}\cdot\boldsymbol{\sigma}^{\mu}\right)_{\alpha,\alpha'} \hat{c}^{\dagger}_{\sigma\mu\alpha}(\mathbf{k}) \hat{c}_{\sigma\mu\alpha'}(\mathbf{k}) \tag{S20}$$

where $\boldsymbol{\sigma}^{\mu} = (\mu\sigma_x, \sigma_y)$ with $\sigma_{x,y}$ are the Pauli matrices and the valley index $\mu = \pm 1$.

In the Hartree approximation by contracting c and d fermion operators separately, we have

$$\hat{H}_{G-S} = \sum_{\sigma,\alpha,\mu} \sum_{\mathbf{k},\mathbf{G}} \widetilde{V}(\mathbf{G}) \sum_{\widetilde{\mathbf{k}},\sigma'} \langle \hat{d}^{\dagger}_{\sigma'}(\widetilde{\mathbf{k}}) \hat{d}_{\sigma'}(\widetilde{\mathbf{k}}) \rangle \, \hat{c}^{\dagger}_{\sigma\mu\alpha}(\mathbf{k}) \hat{c}_{\sigma\mu\alpha}(\mathbf{k}-\mathbf{G}). \tag{S21}$$

Since the long-wavelength charge order state is insulating presumingly with two spin degenerate electrons occupying each supercell, we have

$$\sum_{\widetilde{\mathbf{k}},\sigma'} \langle \hat{d}^{\dagger}_{\sigma'}(\widetilde{\mathbf{k}}) \hat{d}_{\sigma'}(\widetilde{\mathbf{k}}) \rangle = 2N_d. \tag{S22}$$

Writing $\mathbf{k} = \widetilde{\mathbf{k}} + \mathbf{G}$ with $\mathbf{G}$ in the superlattice reciprocal lattice, the final form of the coupling between graphene and insulating substrate used in our calculations reads

$$\hat{H}_{G-S} = \sum_{\sigma,\alpha,\mu} \sum_{\substack{\mathbf{G},\mathbf{Q} \\ \in\{\mathbf{G_i}\}}} \widetilde{U}_d(\mathbf{Q}) \; \hat{c}^{\dagger}_{\sigma\mu\alpha,\mathbf{G}+\mathbf{Q}}(\widetilde{\mathbf{k}}) \hat{c}_{\sigma\mu\alpha,\mathbf{G}}(\widetilde{\mathbf{k}}). \tag{S23}$$

where

$$\widetilde{U}_d(\mathbf{Q}) = \frac{e^2}{\epsilon_0 \epsilon_r \Omega_d} \frac{e^{-|\mathbf{Q}|d}}{|\mathbf{Q}|} \tag{S24}$$

In the meantime, we integrate out the Hamiltonian for insulating substrate H_S [see Eq. (S2)] so that it becomes a constant charge density, which is omitted in our calculations. To wrap up, we get the effective non-interacting Hamiltonian in continuum in the valley μ

$$H_0^{\mu}(\mathbf{r}) = \hbar v_F \mathbf{k}\cdot\boldsymbol{\sigma}^{\mu} + U_d(\mathbf{r}) \tag{S25}$$

where the Fourier component of $U_d(\mathbf{r})$ is precisely $\widetilde{U}_d(\mathbf{G})$ [see Eq. (S24)] with $\mathbf{G}$ in the reciprocal lattice of the underlying insulating substrate's surface superlattice.

As revealed by Eq. (S24), increasing the interlayer distance d would diminish the interlayer Coulomb interaction between the charges of the Wigner crystal at the surface of the substrate and the Dirac electrons in the graphene layer [see Eq. (2) in the main text]. To estimate the e-e interaction effects in graphene with different interlayer distance d, we have calculated the effective fine structure constant of graphene: $\alpha = e^2/4\pi\epsilon_0\epsilon_r\hbar v_F^{\mathrm{SL}}$, where the Fermi velocity v_F^{SL} is the non-interacting Fermi velocity of the graphene being coupled to the Coulomb superlattice potential. Note that v_F^{SL} is reduced compared to the free-standing non-interacting Fermi velocity v_F due to the effects of Coulomb superlattice potential, whose strength depends on the interlayer distance d, the background dielectric constant ϵ_r, and the superlattice constant L_s of the Wigner crystal formed at the surface of the substrate. In Supplementary Figure 1(a), we plot the effective fine structure constant α as a function of the interlayer distance d, and the superlattice constant L_s, with a fixed background dielectric constant $\epsilon_r = 3$. We note that there is small region in the upper left corner where $\alpha \leq \alpha_c = 0.92$, which means that graphene would remain as a semimetal in this region. We further plot α vs. d in Supplementary Figure 1(b), and find that α decreases with the increase of d, and α becomes smaller than the

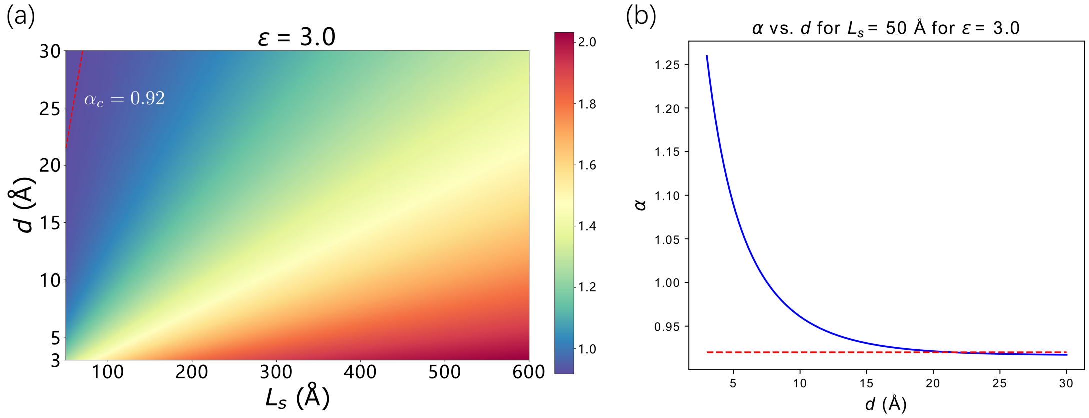

Supplementary Figure 1: (a) Calculated effective fine-structure constant α of graphene in the parameter space of the background Wigner-crystal superlattice L_s, and the interlayer distance between graphene and the substrate d, with the background dielectric constant fixed as $\epsilon_r = 3$. (b) Line plot of α vs. d, with $L_s = 50$ Å, and $\epsilon_r = 3$. The red lines mark the critical value of $\alpha_c = 0.92$ above which the Dirac point of graphene would be gapped by e-e interactions.

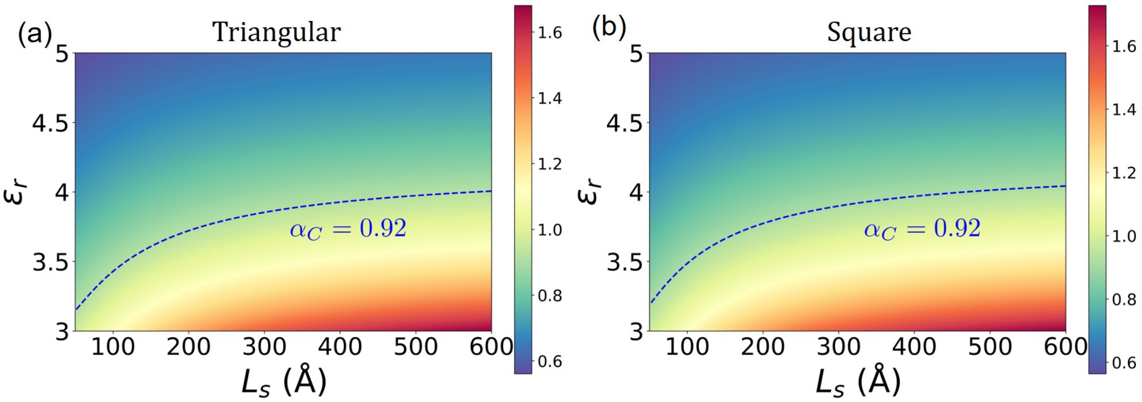

Supplementary Figure 2: Calculated effective fine-structure constant α of graphene in the parameter space of the background Wigner-crystal superlattice L_s, and the interlayer distance between graphene and the substrate d, with the background dielectric constant fixed as $\epsilon_r = 3$, for (a) triangular and (b) square lattice.

critical value 0.92 when $d > 20$ Å, the moment that is expected to undergo a transition from a gapped to a gapless Dirac state. It turns out that the colormaps of α remains qualitatively the same for different lattice geometries, comparing Supplementary Figure 1(a) for rectangular lattice with Supplementary Figure 2 for triangular and square lattice. This also justifies why we can consider only two particular cases, rectangular and triangular lattice, in our work without loss of generality.

In our numerical implementations, the lattice of insulating substrate is set to be rectangular or triangular, from which we obtain qualitatively the same correlated states in the graphene layer. The range of $\{\mathbf{G_i}\}$ is limited to $|n_x|, |n_y| \leq 4$ with $\mathbf{G} = n_x\mathbf{g_x} + n_y\mathbf{g_y}$. $\mathbf{g_{x,y}}$ are the two reciprocal lattice vectors for the rectangular lattice of insulating substrate. The sum over $\mathbf{Q}$ in Eq. (S23) stops at the limit $|n_x| + |n_y| \leq 2$.

SUPPLEMENTARY NOTE 2: TOPOLOGICAL PROPERTIES OF THE NON-INTERACTING HAMILTONIAN

We further study the topological properties of our model Hamiltonian derived in the previous section. Different from magic-angle TBG [1–5], the low-energy subbands for graphene coupled to a rectangular superlattice potential $U_d(\mathbf{r})$ with small anisotropy ($r \sim 1$) turns out to be topologically trivial. To be specific, in Fig. 2d of the main text, we have shown the Berry curvature distribution of the highest valence band of valley K in the mini BZ of the

superlattice with $L_x = 50\,\text{Å}$ and $r = L_y/L_x = 1.2$. We see that the Berry curvature is mostly concentrated at the band crossing points, i.e., the four high symmetry points Γ_s, S_s, X_s, and Y_s. The contributions from the Γ_s and the S_s points are exactly compensated by those from the X_s and Y_s points, resulting in a band with zero Chern number. This is anticipated because the superlattice potential is non-chiral in the sense that it is coupled equally to the two sublattice of graphene. This remains true even including e-e interactions.

Hence, it is unexpected that changing the anisotropy r and the lattice size L_s of the superlattice potential U_d can make the subbands topological. For example, keeping $L_x = 50\,\text{Å}$ but with $r = 3.0$, the valley Chern number of the low-energy subbands become nonzero. For valley K, the highest valence band and lowest conduction band now have a Chern number $C_v = \pm 1$, respectively. As shown in Supplementary Figure 3(a), besides the four high symmetry points, it appears another two "hot spots" (annotated by green circles) along the line connecting Γ_s and X_s. This new contribution breaks the balance between positive and negative contribution of Berry curvature to Chern number, leading to non-zero valley Chern number. Such contribution stems from a new crossing point between the low-energy valence and conduction bands along the k_x-direction through changing merely the anisotropy parameter r, as shown in Supplementary Figure 3(c) with red dot in green circle. From the animated Supplementary Figure 4, we see that while increasing r, the Fermi velocity is gradually reduced, and at $r = 2.7$, becomes vanishingly small along one direction due to Klein tunneling effects. Then, further tuning r germinates a band crossing originated from the Dirac point then gradually moving away from it. Alternatively, we find that changing L_s can also control the valley Chern number of the subbands, since L_s is encoded in the superlattice potential (see Eq. (S24)). For example, with $r = 3$ and $L_s = 600\,\text{Å}$, as shown in Supplementary Figure 3(b), while the highest valence band remains topological with non-zero valley Chern number 1 for valley K with the two aforementioned crossing points (green circles) merely moving to X_s, the lowest conduction band turns out to be topologically trivial. This is due to two new band crossing points (orange circles) close to the Y_s-S_s line between the lowest and the second lowest conduction bands, as annotated by red dots in an orange circle in Supplementary Figure 3(d). Such topologically nontrivial bands are particularly surprising for our system, since the Dirac fermions are coupled to a "trivial" superlattice potential coupled identically on two sublattices, different from the case of magic-angle TBG. Thus, the nontrivial topology must arise by virtue of the intrinsic Berry phases of the Dirac cones.

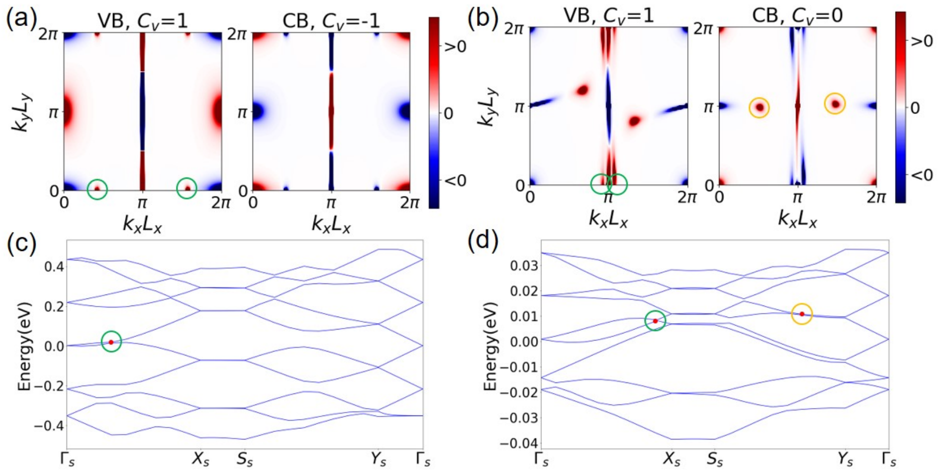

Supplementary Figure 3: (a) and (b) shows the distribution of Berry curvature in the $r = 3$ superlattice's BZ of the lowest valence and conduction band in valley K for $L_s = 50$ and $600\,\text{Å}$, respectively. Their corresponding valley Chern number are also given on the top of each panel. (c) and (d) are the non-interacting band structure of the $r = 3$ superlattice with $L_s = 50$ and $600\,\text{Å}$.

We further show the non-interacting energy spectra and distributions of Berry curvature in the first Brillouin zone for various superlattice constants (L_s) and anisotropy parameters (r), i.e., $L_s = 50$, 200, $600\,\text{Å}$ with $r = 1.2$ and 3. The plots are given in Supplementary Figure 5, 6, 7 for $L_s = 50$, 200, $600\,\text{Å}$, respectively. Since the system preserves time-reversal symmetry and the superlattice potential U_d is diagonal in the sublattice subspace, the non-interacting energy spectrum in valley K' is exactly the same as that in valley K so that we only plot the spectrum for valley K here. As shown below, the distribution of Berry curvature of the highest valence and the lowest conduction band in valley K is exactly opposite to that in valley K' as another consequence of time-reversal symmetry of the system.

We also provide separately six videos in other Supplementary Data, which show the non-interacting energy spectra

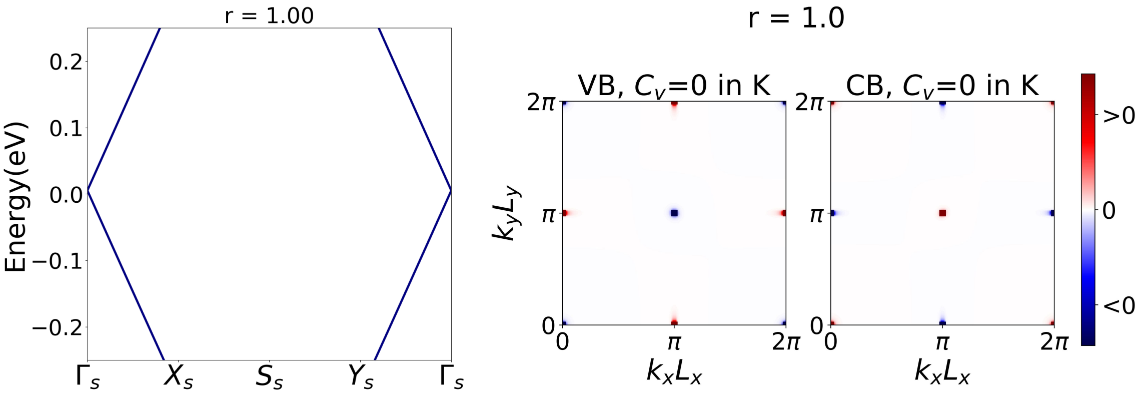

Supplementary Figure 4: Animation (click the figure in, for example, Adobe Acrobat PDF reader to activate) for the non-interacting band structures of graphene on a superlattice with $L_s = 50\,\text{Å}$ and the anisotropy parameter r varying from 1 to 5.

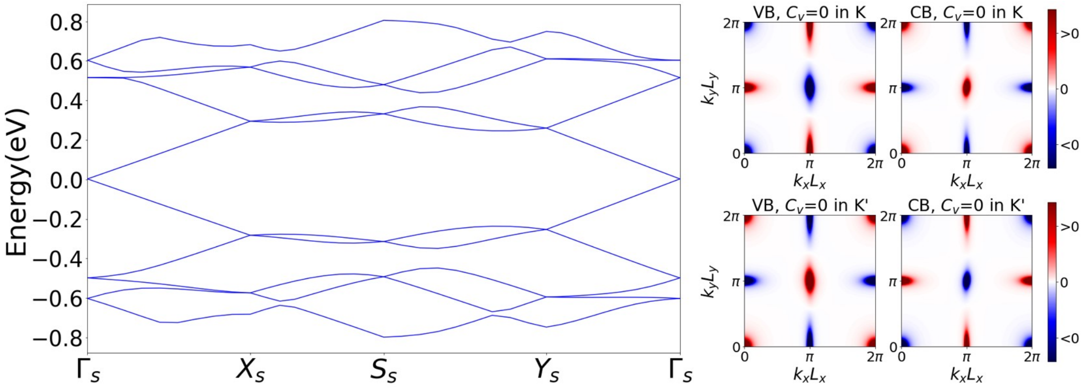

(a)

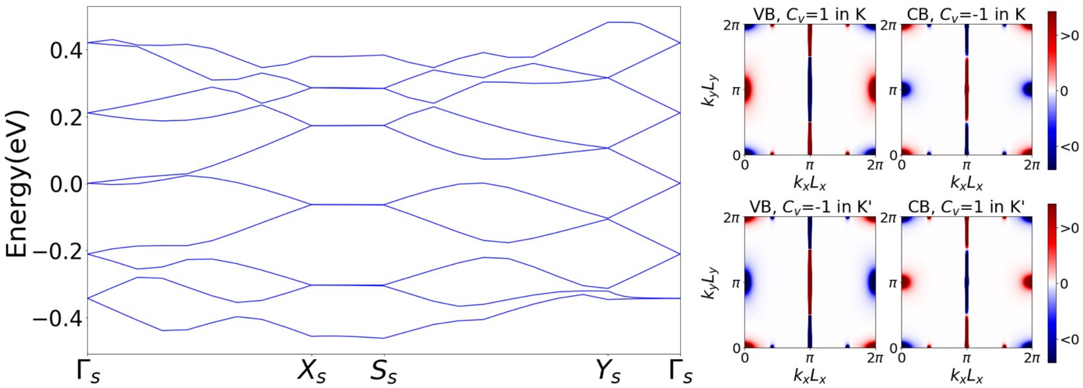

(b)

Supplementary Figure 5: Non-interacting spectrum and distribution of Berry curvature in the first Brillouin zone for $L_s = 50\,\text{Å}$ with (a) $r = 1.2$ and (b) $r = 3$. Please refer to Supplementary Note 2.

and distributions of Berry curvature in the first Brillouin zone for $L_s = 50$, 200, 600 Å with $r =$ 1-10, respectively. We note that the anisotropic charge ordered superlattices may be realized in two ways. First, one can design a spatially modulated electrostatic potential, which has been realized in monolayer graphene by inserting a patterned dielectric superlattice between the gate and the sample [6]. Then, the anisotropy of the superlattice can be artificially tuned by the dielectric patterning in the substrate. Second, for some given carrier density, the Fermi surface of the conduction (or valence) band of the substrate may be (partially) nested, which may lead to a charge density wave (CDW) state

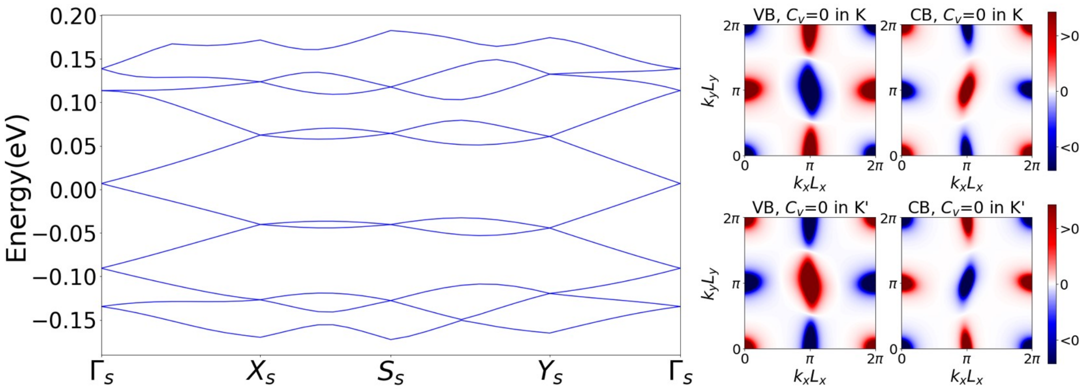

(a)

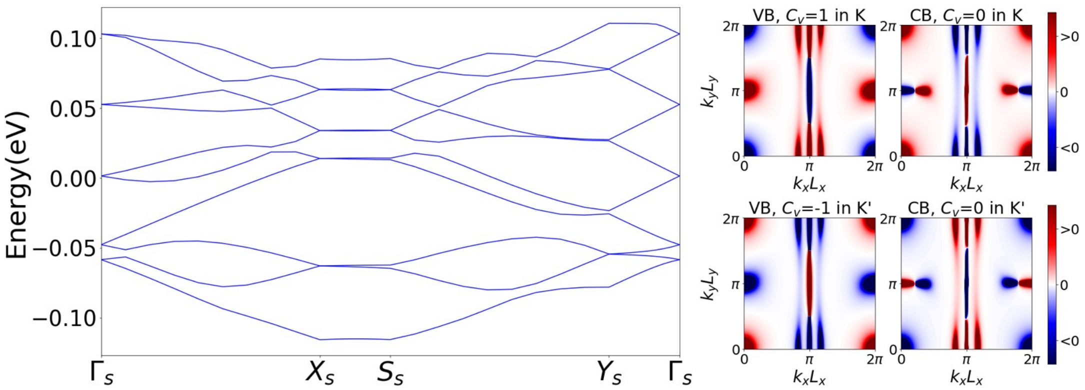

(b)

Supplementary Figure 6: Non-interacting spectrum and distribution of Berry curvature in the first Brillouin zone for $L_s = 200\,\text{Å}$ with (a) $r = 1.2$ and (b) $r = 3$. Please refer to Supplementary Note 2.

with the nesting wavevector. For example, for CrOCl, the Fermi surfaces under different Fermi energies (above the conduction band minimum) are given in Supplementary Figure 15(c). Clearly, under some proper fillings, the Fermi surfaces are nested or partially nested, which may give rise to CDW states with anisotropic superlattices. We note that topologically nontrivial flat bands have also been proposed to exist in Bernal bilayer graphene coupled with a background superlattice potential [7].

SUPPLEMENTARY NOTE 3: RENORMALIZATION GROUP DERIVATIONS

The derivation shown in this section is inspired from Ref. 8. The e-e Coulomb interaction operator in our derivations is written as

$$\hat{V}_{\text{int}} = \frac{1}{2}\int d^2\mathbf{r} d^2\mathbf{r}' V_c(\mathbf{r}-\mathbf{r}')\hat{\rho}(\mathbf{r})\hat{\rho}(\mathbf{r}') \tag{S26}$$

where $V_c(\mathbf{r}) = e^2/4\pi\epsilon_0\epsilon_r r$ and $\hat{\rho}(\mathbf{r})$ is the density operator of electrons at $\mathbf{r}$. The Hamiltonian Eq. (S25) is defined at some high energy cut-off $\pm E_c$. We focus in the valley $\mu = +1$ by the virtue of which the derivation for the valley $\mu = -1$ is immediate and the results are identical. Remember that the parameters v_F and $\widetilde{U}_d(G)$ should be thought of as being fixed by a measurement at E_c without e-e interactions. This also amounts to $\hat{\rho}(\mathbf{r}) = \hat{\psi}^\dagger(\mathbf{r})\hat{\psi}(\mathbf{r})$ with the non-interacting field operator $\hat{\psi}(\mathbf{r})$

$$\hat{\psi}(\mathbf{r}) = \sum_{\substack{\sigma,n,\mathbf{k};\\ |\epsilon_{n,\mathbf{k}}|\leq E_c}} \phi_{\sigma n\mathbf{k}}(\mathbf{r})\hat{c}_{\sigma n}(\mathbf{k}) \tag{S27}$$

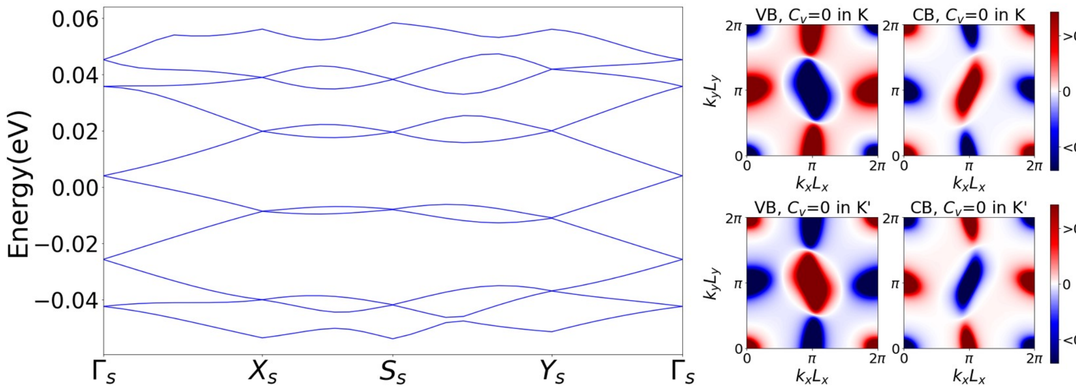

(a)

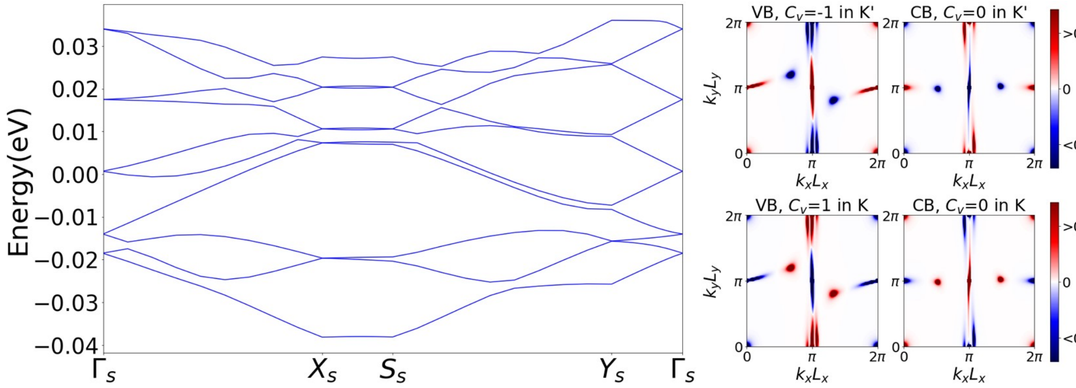

(b)

Supplementary Figure 7: Non-interacting spectrum and distribution of Berry curvature in the first Brillouin zone for $L_s = 600\,\text{Å}$ with (a) $r = 1.2$ and (b) $r = 3$. Please refer to Supplementary Note 2.

where $\phi_{\sigma n\mathbf{k}}(\mathbf{r})$ is the wavefunction of an eigenstate of the non-interacting Hamiltonian H_0 [see Eq. (S25)] with energy $\epsilon_{n,\mathbf{k}}$ and its associated annihilation operator is $\hat{c}_{\sigma n}(\mathbf{k})$.

Electron-electron interaction in a lower energy window

Now we change the cut-off E_c to a smaller one E_c' and see how these parameters are modified by $\hat{V}_{\text{int}}$. $\hat{V}_{\text{int}}$ can be treated perturbatively when E_c' is much larger than any other energy scale in the system. To do so, we split the field operator $\hat{\psi}(\mathbf{r}) = \hat{\psi}^<(\mathbf{r}) + \hat{\psi}^>(\mathbf{r})$ where

$$\hat{\psi}^<(\mathbf{r}) = \sum_{\substack{\sigma,n,\mathbf{k};\\ |\epsilon_{n,\mathbf{k}}|\le E_c'}} \phi_{\sigma n\mathbf{k}}(\mathbf{r})\hat{c}_{\sigma n}(\mathbf{k}) \tag{S28}$$

$$\hat{\psi}^>(\mathbf{r}) = \sum_{\substack{\sigma,n,\mathbf{k};\\ E_c'<|\epsilon_{n,\mathbf{k}}|\le E_c}} \phi_{\sigma n\mathbf{k}}(\mathbf{r})\hat{c}_{\sigma n}(\mathbf{k}). \tag{S29}$$

(S30)

Then, we integrate out the fast modes $\hat{\psi}^>(\mathbf{r})$ in the expansion of $\hat{\rho}(\mathbf{r})\hat{\rho}(\mathbf{r}')$. Note that $\hat{\psi}^>(\mathbf{r})$ and $\hat{\psi}^{>\dagger}(\mathbf{r})$ must appear equal times in each terms of the expansion otherwise it would vanish by taking the non-interacting mean value $\langle\dots\rangle_0$.

Explicitly, these terms are retained up to a constant:

$$\begin{aligned}\hat{\rho}(\mathbf{r})\hat{\rho}(\mathbf{r}') &= \hat{\rho}^{<}(\mathbf{r})\hat{\rho}^{<}(\mathbf{r}')\\ &+ \bar{\rho}^{>}(\mathbf{r})\hat{\psi}^{<\dagger}(\mathbf{r}')\hat{\psi}^{<}(\mathbf{r}') + \bar{\rho}^{>}(\mathbf{r}')\hat{\psi}^{<\dagger}(\mathbf{r})\hat{\psi}^{<}(\mathbf{r})\\ &+ \underbrace{\hat{\psi}^{<\dagger}(\mathbf{r})\ \langle\hat{\psi}^{>}(\mathbf{r})\hat{\psi}^{>\dagger}(\mathbf{r}')\rangle_0\ \hat{\psi}^{<}(\mathbf{r}') + \hat{\psi}^{<}(\mathbf{r})\ \langle\hat{\psi}^{>\dagger}(\mathbf{r})\hat{\psi}^{>}(\mathbf{r}')\rangle_0\ \hat{\psi}^{<\dagger}(\mathbf{r}')}_{(*)}\end{aligned}$$

with

$$\hat{\rho}^{<}(\mathbf{r}) = \hat{\psi}^{<\dagger}(\mathbf{r})\hat{\psi}^{<}(\mathbf{r}) \tag{S31}$$

$$\bar{\rho}^{>}(\mathbf{r}) = \sum_{\substack{\sigma,n,\mathbf{k};\\ E_c'<|\epsilon_{n,\mathbf{k}}|\le E_c}} \phi^*_{\sigma n\mathbf{k}}(\mathbf{r})\phi_{\sigma n\mathbf{k}}(\mathbf{r}). \tag{S32}$$

The first term gives the Coulomb e-e interaction between electrons of the slow modes $\hat{\psi}^{<}(\mathbf{r})$ below the new cut-off E_c'. The second and third term could be omitted if the system has particle-hole (p-h) symmetry as in twisted bilayer graphene [8]. In our system described by Eq. (S25), the first nearest-neighbor coupling in $\widetilde{U}_d(\mathbf{G})$ preserves particle-hole (p-h) symmetry. The p-h symmetry is broken if further-neighbor coupling is included, which is exponentially smaller [see Eq. (S24)]. So, it is legitimate in our RG derivation to neglect such weak p-h asymmetry in order to omit the second and the third term in the expansion.

Then, we evaluate the rest of the terms in the expansion, which represents precisely the correction to H_0 from the fast modes $\hat{\psi}^{>}(\mathbf{r})$ via Coulomb e-e interactions. Let us write

$$\begin{aligned}(*) &= \hat{\psi}^{<\dagger}(\mathbf{r})\left(\sum_{\substack{\sigma,n,\mathbf{k};\\ E_c'<\epsilon_{n,\mathbf{k}}\le E_c}} \phi_{\sigma n\mathbf{k}}(\mathbf{r})\phi^*_{\sigma n\mathbf{k}}(\mathbf{r}')\right)\hat{\psi}^{<}(\mathbf{r}') + \hat{\psi}^{<}(\mathbf{r})\left(\sum_{\substack{\sigma,n,\mathbf{k};\\ -E_c'>\epsilon_{n,\mathbf{k}}\ge -E_c}} \phi^*_{\sigma n\mathbf{k}}(\mathbf{r})\phi_{\sigma n\mathbf{k}}(\mathbf{r}')\right)\hat{\psi}^{<\dagger}(\mathbf{r}')\\ &= \hat{\psi}^{<\dagger}(\mathbf{r})\left(\sum_{\substack{\sigma,n,\mathbf{k};\\ E_c'<\epsilon_{n,\mathbf{k}}\le E_c}} \phi_{\sigma n\mathbf{k}}(\mathbf{r})\phi^*_{\sigma n\mathbf{k}}(\mathbf{r}')\right)\hat{\psi}^{<}(\mathbf{r}') + \hat{\psi}^{<\dagger}(\mathbf{r}')\left(\sum_{\substack{\sigma,n,\mathbf{k};\\ -E_c'>\epsilon_{n,\mathbf{k}}\ge -E_c}} -\phi^*_{\sigma n\mathbf{k}}(\mathbf{r})\phi_{\sigma n\mathbf{k}}(\mathbf{r}')\right)\hat{\psi}^{<}(\mathbf{r})\end{aligned}$$

where the minus sign in the second line comes from the exchange the two fermionic operators and the constant arising from the exchange is omitted. Then, the e-e interaction $\hat{V}_{\text{int}}$ in the lower energy window delimited by E_c' is

$$\hat{V}_{\text{int}} = \frac{1}{2}\int d^2\mathbf{r}d^2\mathbf{r}' V_c(\mathbf{r}-\mathbf{r}')\hat{\rho}^{<}(\mathbf{r})\hat{\rho}^{<}(\mathbf{r}') + \frac{1}{2}\int d^2\mathbf{r}d^2\mathbf{r}' V_c(\mathbf{r}-\mathbf{r}')\hat{\psi}^{<\dagger}(\mathbf{r})\mathcal{F}(\mathbf{r},\mathbf{r}')\hat{\psi}^{<}(\mathbf{r}') \tag{S33}$$

with

$$\mathcal{F}(\mathbf{r},\mathbf{r}') = \sum_{\substack{\sigma,n,\mathbf{k};\\ E_c'<|\epsilon_{n,\mathbf{k}}|\le E_c}} \text{sign}(\epsilon_{n,\mathbf{k}})\phi_{\sigma n\mathbf{k}}(\mathbf{r})\phi^*_{\sigma n\mathbf{k}}(\mathbf{r}'). \tag{S34}$$

Evaluation of the correction to the non-interacting Hamiltonian from the fast modes

In the following, we set $\hbar = 1$ for the simplicity in mathematical expressions. Note that $\mathcal{F}(\mathbf{r},\mathbf{r}')$ has the structure of the residue of the Green's function $\hat{G}(z) = (z-\hat{H}_0)^{-1}$ taking only the valley $\mu = +1$ part in $\hat{H}_0 = \hat{H}_G + \hat{H}_{G-S}$ [see Eqs. (S20) and (S23)], namely

$$\mathcal{F}(\mathbf{r},\mathbf{r}') = \oint_{\mathcal{C}} \frac{dz}{2\pi i}\langle\mathbf{r}|\hat{G}(z)|\mathbf{r}'\rangle \tag{S35}$$

where the contour $\mathcal{C}$ encloses the z-plane real line segment $[-E_c, -E_c']$ in the clockwise, and segment $[E_c', E_c]$ in the counterclockwise, sense. As long as E_c' dominates over all other energy scales such as $\widetilde{U}_d(\mathbf{G_0})$ and $v_F G_0$ with $\mathbf{G_0}$ denoting the primitive reciprocal vector of the underlying superlattice, the dominant contribution to the contour

integral can be evaluated perturbatively using $\hat{G}(z) \approx \hat{G}_0(z) + \hat{G}_0(z)\hat{H}_{G-S}\hat{G}_0(z) + \mathcal{O}\left(\widetilde{U}_d^2(\mathbf{G_0})/E_c'^2, v_F^2 G_0^2/E_c'^2\right)$ with $\hat{G}_0(z) = (z - \hat{H}_G)^{-1}$.

It is easier to calculate the Green's function in the plane wave basis $|\mathbf{k}\rangle$

$$\mathcal{F}(\mathbf{r}, \mathbf{r}') = \int \frac{d^2k d^2k'}{(2\pi)^4} e^{i(\mathbf{k}\cdot\mathbf{r} - \mathbf{k}'\cdot\mathbf{r}')} \oint_{\mathcal{C}} \frac{dz}{2\pi i} \langle \mathbf{k}|\hat{G}(z)|\mathbf{k}'\rangle \tag{S36}$$

with

$$|\mathbf{r}\rangle = \int \frac{d^2k}{(2\pi)^2} e^{-i\mathbf{k}\cdot\mathbf{r}} |\mathbf{k}\rangle \tag{S37}$$

$$\langle \mathbf{r}|\mathbf{r}'\rangle = \delta^{(2)}(\mathbf{r} - \mathbf{r}') \tag{S38}$$

$$\langle \mathbf{k}|\mathbf{k}'\rangle = (2\pi)^2 \delta^{(2)}(\mathbf{k} - \mathbf{k}') \tag{S39}$$

where $\delta^{(2)}(\mathbf{x})$ is the 2D Dirac distribution. In the plane wave basis, the evaluation of Green's functions is straightforward

$$\langle \mathbf{k}|\hat{G}_0(z)|\mathbf{k}'\rangle = (2\pi)^2 \delta^{(2)}(\mathbf{k} - \mathbf{k}') \frac{1}{2} \sum_{\lambda=\pm} \frac{1 - \lambda\frac{\mathbf{k}}{k}\cdot\boldsymbol{\sigma}}{z - \lambda v_F k} \tag{S40}$$

$$\langle \mathbf{k}|\hat{G}_0(z)\hat{H}_{G-S}\hat{G}_0(z)|\mathbf{k}'\rangle = (2\pi)^2 \delta^{(2)}(\mathbf{k} - \mathbf{k}' + \mathbf{G}) \frac{1}{4} \sum_{\mathbf{G}} \widetilde{U}_d(\mathbf{G}) \sum_{\lambda,\lambda'=\pm} \frac{\left(1 - \lambda\frac{\mathbf{k}}{k}\cdot\boldsymbol{\sigma}\right)\left(1 - \lambda'\frac{\mathbf{k}+\mathbf{G}}{|\mathbf{k}+\mathbf{G}|}\cdot\boldsymbol{\sigma}\right)}{(z - \lambda v_F k)(z - \lambda' v_F |\mathbf{k}+\mathbf{G}|)}. \tag{S41}$$

Then, the contour integral can be easily done:

$$\oint_{\mathcal{C}} \frac{dz}{2\pi i} \langle \mathbf{r}|\hat{G}_0(z)|\mathbf{r}'\rangle = \int_{E_c' < v_F k \leq E_c} \frac{d^2k}{(2\pi)^2} e^{i\mathbf{k}\cdot(\mathbf{r}-\mathbf{r}')} \frac{\mathbf{k}}{k}\cdot\boldsymbol{\sigma} \tag{S42}$$

$$\oint_{\mathcal{C}} \frac{dz}{2\pi i} \langle \mathbf{r}|\hat{G}_0(z)\hat{H}_{G-S}\hat{G}_0(z)|\mathbf{r}'\rangle = \int_{E_c' < v_F k \leq E_c} \frac{d^2k}{(2\pi)^2} e^{i\mathbf{k}\cdot(\mathbf{r}-\mathbf{r}') - i\mathbf{G}\cdot\mathbf{r}'} \frac{1}{4} \sum_{\mathbf{G}} \widetilde{U}_d(\mathbf{G}) \mathcal{I}(\mathbf{k}, \mathbf{G}) \tag{S43}$$

$$\mathcal{I}(\mathbf{k}, \mathbf{G}) = \frac{2}{v_F k + v_F |\mathbf{k}+\mathbf{G}|} \left(1 - \frac{\mathbf{k}\cdot(\mathbf{k}+\mathbf{G})}{k|\mathbf{k}+\mathbf{G}|} + \frac{i\sigma_z(\mathbf{k}\times\mathbf{G})\cdot\hat{\mathbf{z}}}{k|\mathbf{k}+\mathbf{G}|}\right). \tag{S44}$$

Renormalization group flow equations

Now we only have to insert the previous results into the second term in Eq. (S33) to derive the RG equations for v_F and $\widetilde{U}_d(\mathbf{G})$. Let us compute first the integral for $\langle \mathbf{r}|\hat{G}_0(z)|\mathbf{r}'\rangle$. After writing the 2D Coulomb potential in Fourier space $\widetilde{V}_{2\mathrm{D}}(\mathbf{q}) = e^2/2\epsilon_0\epsilon_r q$, we have

$$\begin{aligned} &\frac{1}{2}\int d^2\mathbf{r} d^2\mathbf{r}' V_c(\mathbf{r} - \mathbf{r}') \oint_{\mathcal{C}} \hat{\psi}^{<\dagger}(\mathbf{r}) \frac{dz}{2\pi i} \langle \mathbf{r}|\hat{G}_0(z)|\mathbf{r}'\rangle \hat{\psi}^{<}(\mathbf{r}') \\ &= \int \frac{d^2q}{(2\pi)^2} \hat{\tilde{\psi}}^{<\dagger}(\mathbf{q}) \underbrace{\left(\int_{E_c' < v_F k \leq E_c} \frac{d^2k}{(2\pi)^2} \frac{e^2}{4\epsilon_0\epsilon_r |\mathbf{q}-\mathbf{k}|} \frac{\mathbf{k}}{k}\cdot\boldsymbol{\sigma}\right)}_{\text{(A)}} \hat{\tilde{\psi}}^{<}(\mathbf{q}) \end{aligned}$$

with $\hat{\tilde{\psi}}^{<}(\mathbf{q})$ is the Fourier transform of $\hat{\psi}^{<}(\mathbf{r})$. Since $v_F q \ll E_c'$, we can Taylor expand (A) in terms of q/k. The leading order reads

$$\text{(A)} = \frac{e^2}{16\pi\epsilon_0\epsilon_r} \log\left(\frac{E_c}{E_c'}\right) \mathbf{q}\cdot\boldsymbol{\sigma}. \tag{S45}$$

Therefore, the RG equation reads

$$\frac{dv_F}{d\log E_c} = -\frac{e^2}{16\pi\epsilon_0\epsilon_r}. \tag{S46}$$

Actually, we find the famous result of the Fermi velocity renormalization in graphene due to the e-e interactions.

In the same way, we calculate the integral for $\langle \mathbf{r}|\hat{G}_0(z)\hat{H}_{G-S}\hat{G}_0(z)|\mathbf{r}'\rangle$:

$$\begin{aligned}&\frac{1}{2}\int d^2\mathbf{r}d^2\mathbf{r}'V_c(\mathbf{r}-\mathbf{r}')\oint_{\mathcal{C}}\hat{\psi}^{<\dagger}(\mathbf{r})\frac{dz}{2\pi i}\langle\mathbf{r}|\hat{G}_0(z)|\mathbf{r}'\rangle\hat{\psi}^{<}(\mathbf{r}')\\ &=\int\frac{d^2q}{(2\pi)^2}\hat{\tilde{\psi}}^{<\dagger}(\mathbf{q}-\mathbf{G})\underbrace{\left(\int_{E_c'<v_Fk\leq E_c}\frac{d^2k}{(2\pi)^2}\widetilde{V}_{2\mathrm{D}}(\mathbf{q}-\mathbf{k}-\mathbf{G})\frac{1}{8}\sum_{\mathbf{G}}\widetilde{U}_d(\mathbf{G})\mathcal{I}(\mathbf{k},\mathbf{G})\right)}_{\text{(B)}}\hat{\tilde{\psi}}^{<}(\mathbf{q}).\end{aligned}$$

Since $v_Fq, v_FG \ll E_c'$, we can Taylor expand (B) in terms of q/k and G/k (considered as if they have the same order of magnitude). The leading order reads

$$\text{(B)} = \frac{e^2}{16\epsilon_0\epsilon_r}G^2\left(\frac{1}{E_c'}-\frac{1}{E_c}\right)+\mathcal{O}\left(\frac{v_F^3q^3}{E_c'^3},\frac{v_F^3G^3}{E_c'^3}\right), \tag{S47}$$

which can be neglected under the first-order RG procedure, namely

$$\frac{d\widetilde{U}_d(\mathbf{G})}{d\log E_c}=0. \tag{S48}$$

In summary, we have shown that the Fermi velocity in graphene v_F is renormalized by the e-e Coulomb interaction in the standard way while the superlattice potential $U_d(\mathbf{r})$ keep its value unchanged. In our numerical study of e-e interactions, we use the renormalized Fermi velocity v_F^* in the Hartree-Fock calculations, where we have to take a cut-off n_{cut} to the number of bands, to include the contributions from the higher energy bands outside the cut-off. Technically, we use

$$v_F^* = v_F\left(1+\frac{e^2}{16\pi\epsilon_0\epsilon_r v_F}\log\left(\frac{L_s}{n_{\mathrm{cut}}a_0}\right)\right) \tag{S49}$$

where L_s and a_0 are the lattice constant of the superlattice of $U_d(\mathbf{r})$ and the carbon-carbon bond length in graphene, respectively. Here, the ratio $L_s/n_{\mathrm{cut}}a_0$ plays the role of E_c/E_c'.

SUPPLEMENTARY NOTE 4: HARTREE-FOCK APPROXIMATIONS TO ELECTRON-ELECTRON INTERACTIONS

The derivation shown in this section is inspired from Ref. 9. We consider the Coulomb interactions in graphene

$$\hat{V}_{\mathrm{int}}=\frac{1}{2}\int d^2rd^2r'\sum_{\sigma,\sigma'}\hat{\psi}_\sigma^\dagger(\mathbf{r})\hat{\psi}_{\sigma'}^\dagger(\mathbf{r}')V_{\mathrm{int}}(|\mathbf{r}-\mathbf{r}'|)\hat{\psi}_{\sigma'}(\mathbf{r}')\hat{\psi}_\sigma(\mathbf{r}) \tag{S50}$$

where $\hat{\psi}_\sigma(\mathbf{r})$ is real-space electron annihilation operator at $\mathbf{r}$ with spin σ. This interaction can be written as

$$\hat{V}_{\mathrm{int}}=\frac{1}{2}\sum_{ii'jj'}\sum_{\alpha\alpha'\beta\beta'}\sum_{\sigma\sigma'}\hat{c}_{i,\sigma\alpha}^\dagger\hat{c}_{i',\sigma'\alpha'}^\dagger V_{ij,i'j'}^{\alpha\beta\sigma,\alpha'\beta'\sigma'}\hat{c}_{j',\sigma'\beta'}\hat{c}_{j,\sigma\beta}\ , \tag{S51}$$

where

$$\begin{aligned}V_{ij,i'j'}^{\alpha\beta\sigma,\alpha'\beta'\sigma'}=&\int d^2rd^2r'V_{\mathrm{int}}(|\mathbf{r}-\mathbf{r}'|)\,\phi_\alpha^*(\mathbf{r}-\mathbf{R}_i-\tau_\alpha)\,\phi_\beta(\mathbf{r}-\mathbf{R}_j-\tau_\beta)\phi_{\alpha'}^*(\mathbf{r}-\mathbf{R}_i'-\boldsymbol{\tau}_{\alpha'})\phi_{\beta'}(\mathbf{r}-\mathbf{R}_j'-\boldsymbol{\tau}_{\beta'})\\ &\times\chi_\sigma^\dagger\chi_{\sigma'}^\dagger\chi_{\sigma'}\chi_\sigma.\end{aligned} \tag{S52}$$

Here i, α, and σ refer to Bravis lattice vectors, layer/sublattice index, and spin index. ϕ is Wannier function and χ is the two-component spinor wave function. We further assume that the "density-density" like interaction is dominant

in the system, i.e., $V_{ij,i'j'}^{\alpha\beta\sigma,\alpha'\beta'\sigma'} \approx V_{ii,i'i'}^{\alpha\alpha\sigma,\alpha'\alpha'\sigma'} \equiv V_{i\sigma\alpha,i'\sigma'\alpha'}$, then the Coulomb interaction is simplified to

$$\begin{aligned}\hat{V}_{\text{int}} =& \frac{1}{2}\sum_{ii'}\sum_{\alpha\alpha'}\sum_{\sigma\sigma'} \hat{c}^{\dagger}_{i,\sigma\alpha}\hat{c}^{\dagger}_{i',\sigma'\alpha} V_{i\sigma\alpha,i'\sigma'\alpha'}\hat{c}_{i',\sigma'\alpha'}\hat{c}_{i,\sigma\alpha} \\ =& \frac{1}{2}\sum_{i\alpha\neq i'\alpha'}\sum_{\sigma\sigma'} \hat{c}^{\dagger}_{i,\sigma\alpha}\hat{c}^{\dagger}_{i',\sigma'\alpha'} V_{i\alpha,i'\alpha'}\hat{c}_{i',\sigma'\alpha'}\hat{c}_{i,\sigma\alpha} \\ &+ \sum_{i\alpha} U_0 \hat{c}^{\dagger}_{i,\uparrow\alpha}\hat{c}^{\dagger}_{i,\downarrow\alpha}\hat{c}_{i,\downarrow\alpha}\hat{c}_{i,\uparrow\alpha}\end{aligned} \tag{S53}$$

Here we can see that the Coulomb interaction can be divided into intersite Coulomb interaction and on-site Coulomb interaction. Given that the electron density is low (10^{11} cm^{-2}), i.e., a few electrons per supercell, the chance that two electrons meet at the same atomic site is very low. The Coulomb correlations between two electron are mostly contributed by the inter-site Coulomb interactions. Therefore, the on-site Hubbard interaction has been neglected in our calculations.

In order to model the screening effects to the e-e Coulomb interactions from the dielectric environment, we introduce the double-gate screening form of V_{int}, whose Fourier transform is expressed as

$$V_{\text{int}}(\mathbf{q}) = \frac{e^2 \tanh(qd_s)}{2\Omega_0\epsilon_r\epsilon_0 q} \ , \tag{S54}$$

where Ω_0 is the area of the superlattice's primitive cell, ϵ_r is a background dielectric constant and the thickness between two gates is $d_s = 400\,\text{Å}$.

Since we are interested in the low-energy bands, the intersite Coulomb interactions can be divided into the intra-valley term and the inter-valley term. The intra-valley term $\hat{V}^{\text{intra}}$ can be expressed as

$$\hat{V}^{\text{intra}} = \frac{1}{2N_s}\sum_{\alpha\alpha'}\sum_{\mu\mu',\sigma\sigma'}\sum_{\mathbf{k}\mathbf{k}'\mathbf{q}} V_{\text{int}}(\mathbf{q})\,\hat{c}^{\dagger}_{\sigma\mu\alpha}(\mathbf{k}+\mathbf{q})\hat{c}^{\dagger}_{\sigma'\mu'\alpha'}(\mathbf{k}'-\mathbf{q})\hat{c}_{\sigma'\mu'\alpha'}(\mathbf{k}')\hat{c}_{\sigma\mu\alpha}(\mathbf{k}) \ , \tag{S55}$$

with N_s is the total number of the superlattice's sites. The inter-valley term $\hat{V}^{\text{inter}}$ is expressed as

$$\hat{V}^{\text{inter}} = \frac{1}{2N_s}\sum_{\alpha\alpha'}\sum_{\mu,\sigma\sigma'}\sum_{\mathbf{k}\mathbf{k}'\mathbf{q}} V_{\text{int}}(|\mathbf{K}-\mathbf{K}'|)\,\hat{c}^{\dagger}_{\sigma\mu\alpha}(\mathbf{k}+\mathbf{q})\hat{c}^{\dagger}_{\sigma'-\mu\alpha'}(\mathbf{k}'-\mathbf{q})\hat{c}_{\sigma'\mu\alpha'}(\mathbf{k}')\hat{c}_{\sigma-\mu\alpha}(\mathbf{k}) \ . \tag{S56}$$

$\hat{V}^{\text{intra}}$ includes the Coulomb scattering processes of two electrons created and annihilated in the same valley, and $\hat{V}^{\text{inter}}$ includes the processes that two electrons are created in μ and $-\mu$ and get annihilated in $-\mu$ and μ valleys. Here the atomic wavevector $\mathbf{k}$ is expanded around the valley K^{μ} in the big Brillouin zone of graphene, which can be decomposed as $\mathbf{k} = \widetilde{\mathbf{k}} + \mathbf{G}$, where $\widetilde{\mathbf{k}}$ is the superlattice wavevector in the superlattice Brillouin zone, and $\mathbf{G}$ denotes a superlattice reciprocal lattice vector.

The electron annihilation operator can be transformed from the original basis to the band basis:

$$\hat{c}_{\sigma\mu\alpha}(\mathbf{k}) = \sum_n C_{\sigma\mu\alpha\mathbf{G},n}(\widetilde{\mathbf{k}})\,\hat{c}_{\sigma\mu,n}(\widetilde{\mathbf{k}}) \ , \tag{S57}$$

where $C_{\sigma\mu\alpha\mathbf{G},n}(\widetilde{\mathbf{k}})$ is the expansion coefficient in the n-th Bloch eigenstate at $\widetilde{\mathbf{k}}$ of valley μ:

$$|\mu, n; \widetilde{\mathbf{k}}\rangle = \sum_{\alpha\mathbf{G}} C_{\sigma\mu\alpha\mathbf{G},n}(\widetilde{\mathbf{k}})\,|\sigma,\mu,\alpha,\mathbf{G};\widetilde{\mathbf{k}}\rangle \ . \tag{S58}$$

We note that the non-interacting Bloch functions are spin degenerate due to the separate spin rotational symmetry ($SU(2)\otimes SU(2)$ symmetry) of each valley. Using the transformation given in Eq. (S57), the intra- and inter-valley Coulomb interaction can be written in the band basis

$$\begin{aligned}\hat{V}^{\text{intra}} =& \frac{1}{2N_s}\sum_{\widetilde{\mathbf{k}}\widetilde{\mathbf{k}}'\widetilde{\mathbf{q}}}\sum_{\substack{\mu\mu'\\ \sigma\sigma'}}\sum_{\substack{nm\\ n'm'}}\left(\sum_{\mathbf{Q}} V_{\text{int}}(\mathbf{Q}+\widetilde{\mathbf{q}})\,\Omega^{\mu\sigma,\mu'\sigma'}_{nm,n'm'}(\widetilde{\mathbf{k}},\widetilde{\mathbf{k}}',\widetilde{\mathbf{q}},\mathbf{Q})\right) \\ &\times \hat{c}^{\dagger}_{\sigma\mu,n}(\widetilde{\mathbf{k}}+\widetilde{\mathbf{q}})\hat{c}^{\dagger}_{\sigma'\mu',n'}(\widetilde{\mathbf{k}}'-\widetilde{\mathbf{q}})\hat{c}_{\sigma'\mu',m'}(\widetilde{\mathbf{k}}')\hat{c}_{\sigma\mu,m}(\widetilde{\mathbf{k}})\end{aligned} \tag{S59}$$

and

$$\hat{V}^{\rm inter} = \frac{1}{2N_s}\sum_{\widetilde{\mathbf{k}}\widetilde{\mathbf{k}}'\widetilde{\mathbf{q}}}\sum_{\substack{\sigma\sigma'\\ \mu}}\sum_{\substack{nm\\ n'm'}}\left(\sum_{\mathbf{Q}} V_{\rm int}(|\mathbf{K}-\mathbf{K}'|)\,\widetilde{\Omega}^{\mu,\sigma\sigma'}_{nm,n'm'}(\widetilde{\mathbf{k}},\widetilde{\mathbf{k}}',\widetilde{\mathbf{q}},\mathbf{Q})\right) \times \hat{c}^\dagger_{\sigma\mu,n}(\widetilde{\mathbf{k}}+\widetilde{\mathbf{q}})\hat{c}^\dagger_{\sigma'-\mu,n'}(\widetilde{\mathbf{k}}'-\widetilde{\mathbf{q}})\hat{c}_{\sigma'\mu,m'}(\widetilde{\mathbf{k}}')\hat{c}_{\sigma-\mu,m}(\widetilde{\mathbf{k}}) \tag{S60}$$

where the form factors $\Omega^{\mu\sigma,\mu'\sigma'}_{nm,n'm'}$ and $\widetilde{\Omega}^{\mu,\sigma\sigma'}_{nm,n'm'}$ are written respectively as

$$\Omega^{\mu\sigma,\mu'\sigma'}_{nm,n'm'}(\widetilde{\mathbf{k}},\widetilde{\mathbf{k}}',\widetilde{\mathbf{q}},\mathbf{Q}) = \sum_{\alpha\alpha'\mathbf{G}\mathbf{G}'} C^*_{\sigma\mu\alpha\mathbf{G}+\mathbf{Q},n}(\widetilde{\mathbf{k}}+\widetilde{\mathbf{q}})C^*_{\sigma'\mu'\alpha'\mathbf{G}'-\mathbf{Q},n'}(\widetilde{\mathbf{k}}'-\widetilde{\mathbf{q}})C_{\sigma'\mu'\alpha'\mathbf{G}',m'}(\widetilde{\mathbf{k}}')C_{\sigma\mu\alpha\mathbf{G},m}(\widetilde{\mathbf{k}}) \tag{S61}$$

and

$$\widetilde{\Omega}^{\mu,\sigma\sigma'}_{nm,n'm'}(\widetilde{\mathbf{k}},\widetilde{\mathbf{k}}',\widetilde{\mathbf{q}},\mathbf{Q}) = \sum_{\alpha\alpha'\mathbf{G}\mathbf{G}'} C^*_{\sigma\mu\alpha\mathbf{G}+\mathbf{Q},n}(\widetilde{\mathbf{k}}+\widetilde{\mathbf{q}})C^*_{\sigma'-\mu\alpha'\mathbf{G}'-\mathbf{Q},n'}(\widetilde{\mathbf{k}}'-\widetilde{\mathbf{q}})C_{\sigma'\mu\alpha'\mathbf{G}',m'}(\widetilde{\mathbf{k}}')C_{\sigma-\mu\alpha\mathbf{G},m}(\widetilde{\mathbf{k}})\,. \tag{S62}$$

We make Hartree-Fock approximation to Eq. (S59) and Eq. (S60) such that the two-particle Hamiltonian is decomposed into a superposition of the Hartree and Fock single-particle Hamiltonians, where the Hartree term is expressed as

$$\hat{V}^{\rm intra}_H = \frac{1}{2N_s}\sum_{\widetilde{\mathbf{k}}\widetilde{\mathbf{k}}'}\sum_{\substack{\mu\mu',\\ \sigma\sigma'}}\sum_{\substack{nm\\ n'm'}}\left(\sum_{\mathbf{Q}} V_{\rm int}(\mathbf{Q})\,\Omega^{\mu\sigma,\mu'\sigma'}_{nm,n'm'}(\widetilde{\mathbf{k}},\widetilde{\mathbf{k}}',0,\mathbf{Q})\right) \times \left(\langle\hat{c}^\dagger_{\sigma\mu,n}(\widetilde{\mathbf{k}})\hat{c}_{\sigma\mu,m}(\widetilde{\mathbf{k}})\rangle\hat{c}^\dagger_{\sigma'\mu',n'}(\widetilde{\mathbf{k}}')\hat{c}_{\sigma'\mu',m'}(\widetilde{\mathbf{k}}') + \langle\hat{c}^\dagger_{\sigma'\mu',n'}(\widetilde{\mathbf{k}}')\hat{c}_{\sigma'\mu',m'}(\widetilde{\mathbf{k}}')\rangle\hat{c}^\dagger_{\sigma\mu,n}(\widetilde{\mathbf{k}})\hat{c}_{\sigma\mu,m}(\widetilde{\mathbf{k}})\right) \tag{S63}$$

and

$$\hat{V}^{\rm inter}_H = \frac{1}{2N_s}\sum_{\widetilde{\mathbf{k}}\widetilde{\mathbf{k}}'}\sum_{\substack{\sigma\sigma'\\ \mu}}\sum_{\substack{nm\\ n'm'}}\left(\sum_{\mathbf{Q}} V_{\rm int}(|\mathbf{K}-\mathbf{K}'|)\,\widetilde{\Omega}^{\mu,\sigma\sigma'}_{nm,n'm'}(\widetilde{\mathbf{k}},\widetilde{\mathbf{k}}',0,\mathbf{Q})\right) \times \left(\langle\hat{c}^\dagger_{\sigma\mu,n}(\widetilde{\mathbf{k}})\hat{c}_{\sigma-\mu,m}(\widetilde{\mathbf{k}})\rangle\hat{c}^\dagger_{\sigma'-\mu,n'}(\widetilde{\mathbf{k}}')\hat{c}_{\sigma'\mu,m'}(\widetilde{\mathbf{k}}') + \langle\hat{c}^\dagger_{\sigma'-\mu,n'}(\widetilde{\mathbf{k}}')\hat{c}_{\sigma'\mu,m'}(\widetilde{\mathbf{k}}')\rangle\hat{c}^\dagger_{\sigma\mu,n}(\widetilde{\mathbf{k}})\hat{c}_{\sigma-\mu,m}(\widetilde{\mathbf{k}})\right)\,. \tag{S64}$$

The Fock term is expressed as:

$$\hat{V}^{\rm intra}_F = -\frac{1}{2N_s}\sum_{\widetilde{\mathbf{k}}\widetilde{\mathbf{k}}'}\sum_{\substack{\mu\mu',\\ \sigma\sigma'}}\sum_{\substack{nm\\ n'm'}}\left(\sum_{\mathbf{Q}} V_{\rm int}(\widetilde{\mathbf{k}}'-\widetilde{\mathbf{k}}+\mathbf{Q})\,\Omega^{\mu\sigma,\mu'\sigma'}_{nm,n'm'}(\widetilde{\mathbf{k}},\widetilde{\mathbf{k}}',\widetilde{\mathbf{k}}'-\widetilde{\mathbf{k}},\mathbf{Q})\right) \times \left(\langle\hat{c}^\dagger_{\sigma\mu,n}(\widetilde{\mathbf{k}}')\hat{c}_{\sigma'\mu',m'}(\widetilde{\mathbf{k}}')\rangle\hat{c}^\dagger_{\sigma'\mu',n'}(\widetilde{\mathbf{k}})\hat{c}_{\sigma\mu,m}(\widetilde{\mathbf{k}}) + \langle\hat{c}^\dagger_{\sigma'\mu',n'}(\widetilde{\mathbf{k}})\hat{c}_{\sigma\mu,m}(\widetilde{\mathbf{k}})\rangle\hat{c}^\dagger_{\sigma\mu,n}(\widetilde{\mathbf{k}}')\hat{c}_{\sigma'\mu',m'}(\widetilde{\mathbf{k}}')\right)\,.$$

and

$$\hat{V}^{\rm inter}_F = -\frac{1}{2N_s}\sum_{\widetilde{\mathbf{k}}\widetilde{\mathbf{k}}'}\sum_{\substack{\sigma\sigma'\\ \mu}}\sum_{\substack{nm\\ n'm'}}\left(\sum_{\mathbf{Q}} V_{\rm int}(|\mathbf{K}-\mathbf{K}'|)\,\widetilde{\Omega}^{\mu,\sigma\sigma'}_{nm,n'm'}(\widetilde{\mathbf{k}},\widetilde{\mathbf{k}}',\widetilde{\mathbf{k}}'-\widetilde{\mathbf{k}},\mathbf{Q})\right) \times \left(\langle\hat{c}^\dagger_{\sigma\mu,n}(\widetilde{\mathbf{k}}')\hat{c}_{\sigma'\mu,m'}(\widetilde{\mathbf{k}}')\rangle\hat{c}^\dagger_{\sigma'-\mu,n'}(\widetilde{\mathbf{k}})\hat{c}_{\sigma-\mu,m}(\widetilde{\mathbf{k}}) + \langle\hat{c}^\dagger_{\sigma'-\mu,n'}(\widetilde{\mathbf{k}})\hat{c}_{\sigma-\mu,m}(\widetilde{\mathbf{k}})\rangle\hat{c}^\dagger_{\sigma\mu,n}(\widetilde{\mathbf{k}}')\hat{c}_{\sigma'\mu,m'}(\widetilde{\mathbf{k}}')\right)\,.$$

We note that the typical intravalley interaction energy $\sim 240\,$meV for $L_s = 50\,$Å and $\epsilon_r = 3$; while the intervalley interaction $\sim 30\,$meV, which is one order of magnitudes smaller than the intravalley interaction, thus we neglect the intervalley term [see Eq. (S56)] in most of our calculations. We also check *a posteriori* that the intervalley Hartree and Fock energies are at least two orders of magnitude smaller than their intravalley counterpart. However, the intervalley interaction is crucial to lift the degeneracy between many-body ground state, as shown in the following section.

SUPPLEMENTARY NOTE 5: RESULTS OF HARTREE-FOCK CALCULATIONS

In this section, we gather the results of Hartree-Fock calculations including Hartree-Fock single-particle spectra and distributions of Berry curvature in the first Brillouin zone for $L_s = 50$, 200, 600 Å.

First, we show the Hartree-Fock single-particle spectrum with a superlattice potential with $r = 1.2$ of $L_s = 50$, 200, 600 Å in Supplementary Figure 8. Here, we use $n_{\text{cut}} = 5$ and study three types of doping: CNP ($\nu = 0$), slight hole doping ($\nu = -0.003$) and slight electron doping ($\nu = +0.003$). As you can see from Supplementary Table 1 and the Hartree-Fock single-particle spectra, the results of a slightly electron-doped system is similar to those for a slightly hole-doped one. Note that we include only intravalley Coulomb interactions in these calculations. As shown in the following, the role of intervalley Coulomb interactions is merely to lift the ground state degeneracy and favor the σ_z-state.

Supplementary Table 1: Parameters extracted from the Hartree-Fock single-particle spectra: gap opened at the CNP ($\nu = 0$) and the ratio between interaction-renormalized Fermi velocity v_F^* and the non-interacting one v_F for different $L_s = 50$, 200, 600 Å with fixed $r = 1.2$.

L_s(Å)	50	200	600
Gap at $\nu = 0.0$ (meV)	17	1.7	0.15
v_F^*/v_F at $\nu = -0.003$	2.1	1.8	1.7
v_F^*/v_F at $\nu = +0.003$	2.1	1.8	1.7

Then, we show in Supplementary Figure 9 the distributions of Berry curvature in the first Brillouin zone of $r = 1.2$ for $L_s = 50$, 200, 600 Å. Here, $n_{\text{cut}} = 5$.

We further calculate the interacting electronic structure of graphene at different filling factors (denoted by ν) away from the CNP, as presented in Supplementary Figure 10. We find that the ground state is generally gapless at nonzero fillings, and the Fermi velocity enhances as the chemical potential approaches the charge neutrality point ($|\nu| \to 0$). In particular, the Fermi velocity increases from $v_F^* = 1.9v_F$ at $\nu = -0.04$, to $v_F^* = 2.2v_F$ at $\nu = -0.003$.

Now we show the effect of intervalley Coulomb interactions by comparing the total energy of σ_z-state with $\tau_z\sigma_z$-state for different $L_s = 50$, 200, 600 Å with fixed $r = 1.2$. We calculate the difference (always negative) between them and see how it changes when we include the intervalley Coulomb interactions. Here, we use $n_{\text{cut}} = 3$.

As you can see from Supplementary Table 2, the energy difference between the total energy of σ_z-state and $\tau_z\sigma_z$-state is enhanced by two orders of magnitude for $L_s = 50$ and 200 Å. However, the energy difference for $L_s = 600$ Å does not benefit anything from intervalley interactions.

Supplementary Table 2: Difference between the total energy of the σ_z-state and $\tau_z\sigma_z$-state, with or without intervalley interactions, for $L_s = 50$, 200, 600 Å with fixed $r = 1.2$.

L_s(Å)	50	200	600
ΔE with only intravalley (μeV)	-0.008	-0.005	-0.05
ΔE with intra- and inter-valley (μeV)	-1.6	-0.1	-0.03

We also have performed Hartree-Fock calculations on a triangular lattice including three valence and three conduction bands ($n_{\text{cut}} = 3$) for $L_s = 50$, 200, 600 Å using 18×18 k-mesh in the BZ. As shown in Supplementary Table 3, the results on a triangular lattice are qualitatively the same as those on a rectangular lattice. This ensures that our conclusions are lattice-independent.

Supplementary Table 3: Parameters extracted from the Hartree-Fock single-particle spectra on a triangular lattice: gap opened at the CNP ($\nu = 0$) and the ratio between interaction-renormalized Fermi velocity v_F^* and the non-interacting one v_F for different $L_s = 50$, 200, 600 Å.

L_s(Å)	50	200	600
Gap at $\nu = 0.0$ (meV)	21	1.9	0.24
v_F^*/v_F at $\nu = -0.003$	2.2	1.7	1.7

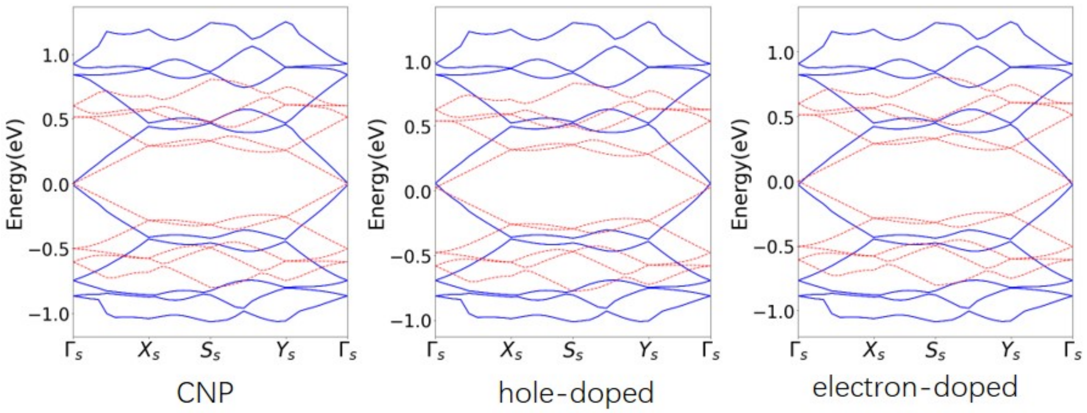

(a)

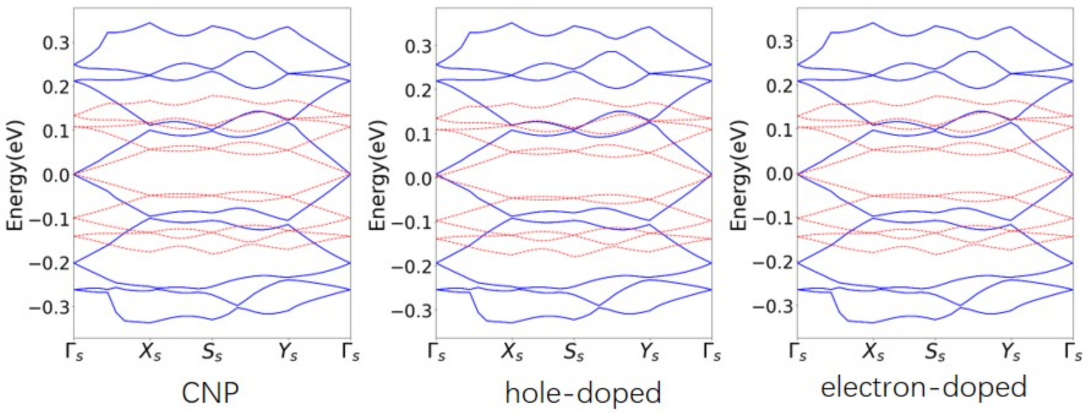

(b)

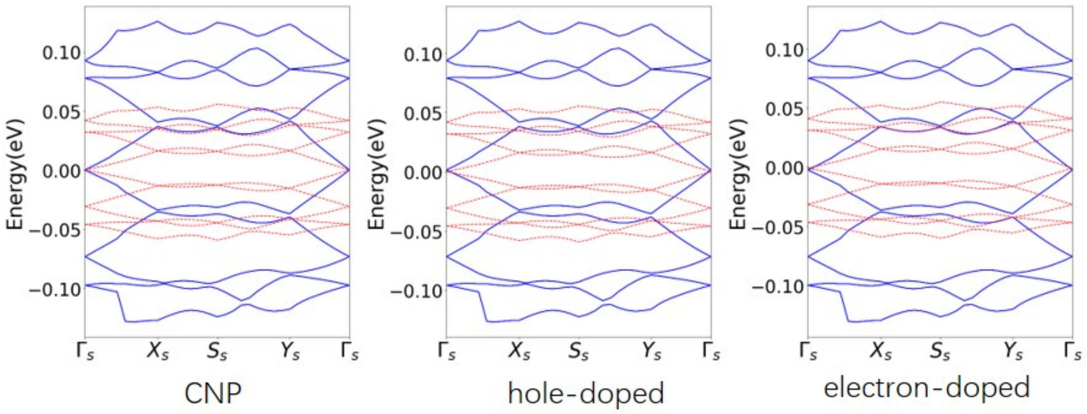

(c)

Supplementary Figure 8: Hartree-Fock single-particle spectra for three different dopings with $r = 1.2$ for (a) $L_s = 50\,\text{Å}$, (b) $L_s = 200\,\text{Å}$ and (c) $L_s = 600\,\text{Å}$.

In some material, there are several valleys to accommodate the charges transferred from graphene so that we need to consider this valley degeneracy in the superlattice potential as follows:

$$U_d(\mathbf{Q}) = e^2 g_v \frac{n(\mathbf{Q}) e^{-|\mathbf{Q}|d}}{2\epsilon_0 \epsilon_r \Omega_0 |\mathbf{Q}|} \tag{S65}$$

where $n(\mathbf{Q})$ is the Fourier transformed charge density per valley at superlattice's reciprocal vector $\mathbf{Q}$ and the valley

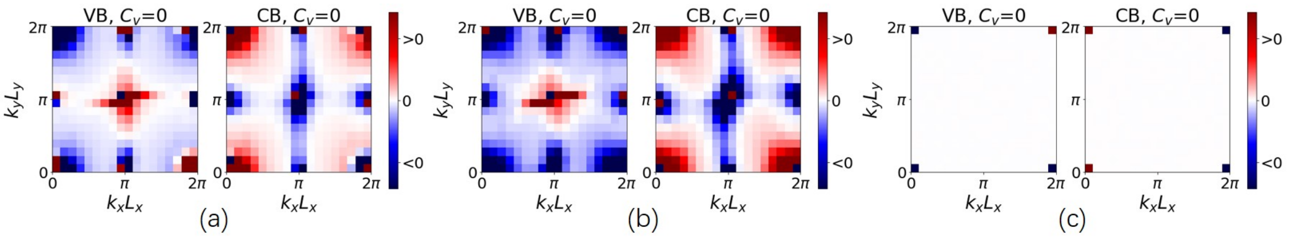

Supplementary Figure 9: Distributions of Berry curvature in the first Brillouin zone of $r = 1.2$ for $L_s =$ (a) 50 Å, (b) 200 Å, (c) 600 Å

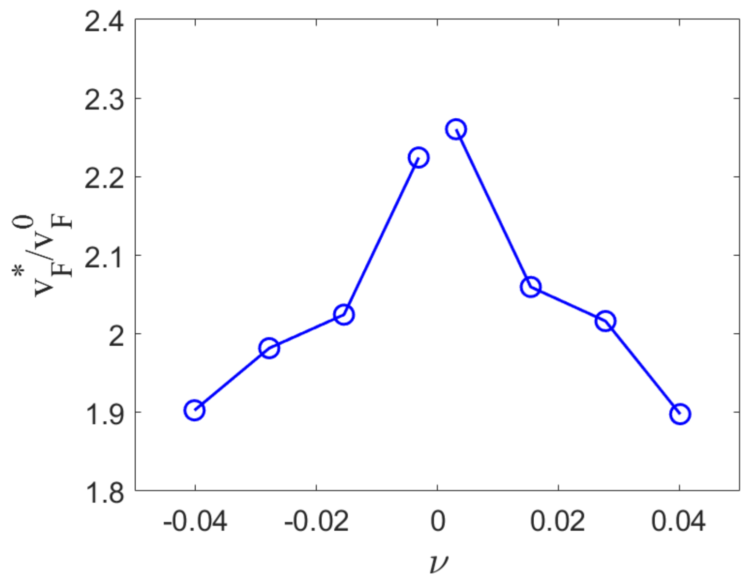

Supplementary Figure 10: The ratio between the interacting Fermi velocity (v_F^*) and the non-interacting one of free-standing graphene (v_F^0) *vs.* the filling factor (ν) with the superlattice constant $L_s = 50$ Å, and a background dielectric constant $\epsilon_r = 3$.

degeneracy $g_v = 2$ for CrOCl. After including this valley degeneracy, our theoretical results are quantitatively consistent with the experimental data, as shown in Supplementary Figure 11. More details on experimental measurements are given in the last section Supplementary Note 8.

SUPPLEMENTARY NOTE 6: GENERAL COUPLED BILAYER SYSTEM IN GRAPHENE-INSULATOR HETEROSTRUCTURES

Previous parts have already proved that the presence of a superlattice potential underneath monolayer graphene sheet helps the latter to open a gap at the CNP, and concomitantly enhance the Fermi velocity around the Dirac point. Now we would like to reconsider the assumptions made for the purpose of writing a simplistic Hamiltonian of our coupled bilayer system given by Eqs. (S1),(S2), and (S3).

Hamiltonians

First, when the Fermi level in graphene aligns closely to the band edge (say, the conduction band minimum) of the insulating substrate, the density of electrons in the conduction band has been assumed to be so low that electrons spontaneously break translational symmetry and form a charge-ordered superlattice. Second, we have assumed that

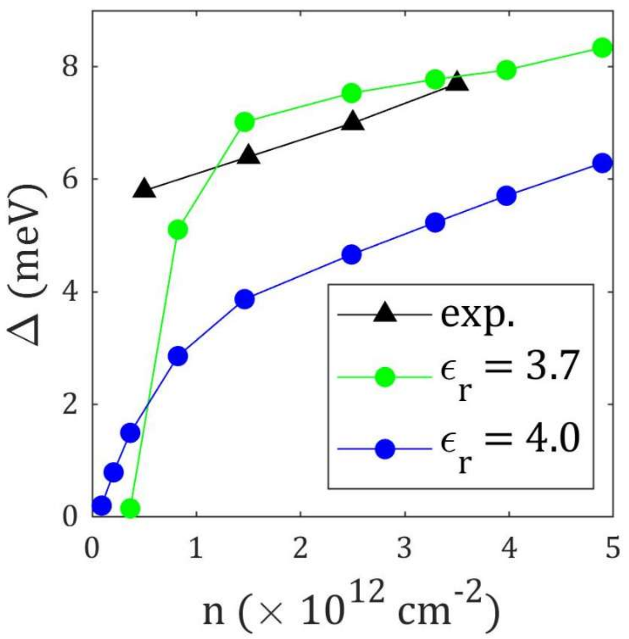

Supplementary Figure 11: Comparison between the experimentally measured gap and the theoretically calculated gaps in graphene-CrOCl heterostructure.

the quantum degrees of freedom on the substrate are completely frozen so that the only effect (other than dielectric screening) acting by substrate to electrons in graphene is to apply a superlattice potential via the long-range Coulomb interactions, stemming from a non-uniform charge density of the charge-ordered state. Lastly, we have drastically supposed that the Wannier functions and the distribution of charge density at the surface of the substrate is as localized as a Dirac-δ-function like distribution in real space [see Eq. (S11)]. These assumptions were good enough if we focus on the physics on the graphene side. In particular, it turns out that the assumption on the extreme localization of the charges and/or Wannier functions [see Eq. (S11)] in the substrate is a good starting point. This is because more extended charge distributions only make the Fourier components of the superlattice potential weaker (to be discussed in the following), which would not change our conclusion qualitatively. However, if we are interested in especially the synergistic interplay between graphene and the insulating substrate, we have to consider integrally the graphene-insulator heterostructure as a coupled bilayer system, and treat both layers on equal footing.

In this section, we aim to study, to the best of our efforts, the coupled bilayer system as a whole, and to treat quantum mechanically the electrons both in the graphene layer and the substrate layer. Most of the above assumptions have to be discarded or replaced by a less harsh one. To this end, in addition to the kinetic energy Eq. (S1) and the long-range intralayer Coulomb interactions of graphene Eq. (S50), we also need to consider the interacting many-body Hamiltonian for electrons on the surface of the substrate. Without loss of generality, we consider the situation that the conduction band minimum (CBM) of the substrate is charge doped.

Specifically, the non-interacting kinetic Hamiltonian of the low-energy electrons around the CBM of the substrate can be modeled by the following one

$$H_s^0 = \sum_{\mathbf{k}_s,\sigma} \left(\frac{\hbar^2 k_s^2}{2m^*} + E_{\mathrm{CBM}} \right) \hat{d}_\sigma^\dagger(\mathbf{k}_s)\, \hat{d}_\sigma(\mathbf{k}_s)\ , \tag{S66}$$

where E_{CBM} is the energy position of CBM with respect to the Fermi level of graphene, and m^* is the effective mass. For CrOCl, $m^* \approx 1.3 m_e$. $\hat{d}_\sigma^{(\dagger)}(\mathbf{k}_s)$ is the annihilation (creation) operator of the low-energy electrons doped to the surface of the substrate, with the wavevector $\mathbf{k}_s$ (expanded around the CBM) in the atomic Brillouin zone of the substrate and spin index σ. Then we consider the long-range Coulomb interactions between electrons at the surface of the substrate:

$$H_{\mathrm{sub}}^{\mathrm{int}} = \frac{1}{2N_s\Omega_s} \sum_{\mathbf{k}_s,\mathbf{k}'_s,\mathbf{q}_s} \sum_{\sigma,\sigma'} V_{\mathrm{int}}(\mathbf{q}_s)\, \hat{d}_\sigma^\dagger(\mathbf{k}_s+\mathbf{q}_s)\, \hat{d}_{\sigma'}^\dagger(\mathbf{k}'_s-\mathbf{q}_s)\, \hat{d}_{\sigma'}(\mathbf{k}'_s)\, \hat{d}_\sigma(\mathbf{k}_s)\ , \tag{S67}$$

where N_s is the total number of *atomic lattice sites* in the substrate layer, Ω_s is the area of the atomic primitive cell in the substrate layer, and $\mathbf{k}_s, \mathbf{k}'_s, \mathbf{q}_s$, denote the atomic wavevectors in the Brillouin zone of the atomic lattice of the substrate. The Coulomb potential $V_{\text{int}}(\mathbf{q})$ is given by Eq. (S54).

We continue to address the interlayer Coulomb interaction, i.e., Eq. (S3). We first transform Eq. (S3) to *the basis of Wannier functions of the atomic lattices of graphene and the substrate* using the following transformations of the field operators:

$$\hat{\psi}^{\dagger}_{c,\sigma}(\mathbf{r}) = \sum_{i,\alpha} \phi^*_{\alpha}(\mathbf{r} - \mathbf{a}_i - \boldsymbol{\tau}_{\alpha})\chi^{\dagger}_{\sigma}\hat{c}^{\dagger}_{i,\sigma\alpha} \tag{S68}$$

$$\hat{\psi}^{\dagger}_{d,\sigma}(\mathbf{r}) = \sum_{i,\alpha} \widetilde{\phi}^*(\mathbf{r} - \mathbf{t}_i)\chi^{\dagger}_{\sigma}\hat{d}^{\dagger}_{i,\sigma} \tag{S69}$$

where $\mathbf{t}_i$ denotes the *atomic lattice sites* of the substrate, which is different from the presumed superlattice sites $\mathbf{R}_i$ in Eq. (S5). $\widetilde{\phi}(\mathbf{r}-\mathbf{t}_i)$ and $\phi^*_{\alpha}(\mathbf{r}-\mathbf{a}_i-\boldsymbol{\tau}_{\alpha})$ denote the atomic-scale Wannier functions for the electrons in the substrate and in the graphene layer, respectively. As already mentioned around Eq. (S5), $\mathbf{a}_i$ denotes the atomic lattice sites of graphene, and $\boldsymbol{\tau}_{\alpha}$ is the position of sublattice α. Using Eq. (S69), Eq. (S3) can be expressed in the atomic Wannier function basis as follows

$$H_{\text{gr-sub}} = \sum_{ij,\alpha}\sum_{\sigma,\sigma'} V(|\mathbf{a}_i + \boldsymbol{\tau}_{\alpha} - \mathbf{t}_j + d\hat{\mathbf{z}}|)\, \hat{c}^{\dagger}_{i,\alpha\sigma}\, \hat{d}^{\dagger}_{j,\sigma'}\, \hat{d}_{j,\sigma'}\, \hat{c}_{i,\alpha\sigma} \tag{S70}$$

where $V(\mathbf{r})$ is the 3D Coulomb potential. Here, we still use approximations similar to Eq. (S11) to get this expression except that we now work on the atomic lattice sites for substrate.

One continues to perform the Fourier transform

$$\hat{c}_{i,\alpha\sigma} = \frac{1}{\sqrt{N_c}}\sum_{\mathbf{k}} e^{i\mathbf{k}\cdot(\mathbf{a}_i+\boldsymbol{\tau}_{\alpha})}\,\hat{c}_{\sigma\alpha}(\mathbf{k})$$

$$\hat{d}_{j,\sigma} = \frac{1}{\sqrt{N_s}}\sum_{\mathbf{k}_s} e^{i\mathbf{k}_s\cdot\mathbf{t}_j}\,\hat{d}_{\sigma}(\mathbf{k}_s)\ , \tag{S71}$$

Then the interlayer Coulomb interaction becomes

$$\begin{aligned} &H_{\text{gr-sub}} \\ =&\frac{1}{N_c N_s}\sum_{ij\alpha}\sum_{\sigma\sigma'}\sum_{\mathbf{k},\mathbf{k}',\mathbf{k}_s,\mathbf{k}'_s} V(|\mathbf{a}_i + \boldsymbol{\tau}_{\alpha} - \mathbf{t}_j + d\hat{\mathbf{z}}|)\, e^{i(\mathbf{k}-\mathbf{k}')\cdot(\mathbf{a}_i+\boldsymbol{\tau}_{\alpha})}\, e^{i(\mathbf{k}_s-\mathbf{k}'_s)\cdot\mathbf{t}_j}\, \hat{c}^{\dagger}_{\sigma\alpha}(\mathbf{k})\, \hat{d}^{\dagger}_{\sigma'}(\mathbf{k}_s)\, \hat{d}_{\sigma'}(\mathbf{k}'_s)\, \hat{c}_{\sigma\alpha}(\mathbf{k}') \\ =&\frac{1}{N_c N_s}\sum_{ij\alpha}\sum_{\sigma\sigma'}\sum_{\mathbf{k},\mathbf{k}',\mathbf{k}_s,\mathbf{k}'_s} V(|\mathbf{a}_i + \boldsymbol{\tau}_{\alpha} - \mathbf{t}_j + d\hat{\mathbf{z}}|)\, e^{i(\mathbf{k}-\mathbf{k}')\cdot(\mathbf{a}_i+\boldsymbol{\tau}_{\alpha}-\mathbf{t}_j)}\, e^{i(\mathbf{k}_s-\mathbf{k}'_s+\mathbf{k}-\mathbf{k}')\cdot\mathbf{t}_j}\, \hat{c}^{\dagger}_{\sigma\alpha}(\mathbf{k})\, \hat{d}^{\dagger}_{\sigma'}(\mathbf{k}_s)\, \hat{d}_{\sigma'}(\mathbf{k}'_s)\, \hat{c}_{\sigma\alpha}(\mathbf{k}')\ . \end{aligned} \tag{S72}$$

Let $\mathbf{k}' - \mathbf{k} = \mathbf{q}_s + \mathbf{g}_s$, where $\mathbf{q}_s$ is a wavevector within the substrate's Brillouin zone, and $\mathbf{g}_s$ is the corresponding reciprocal vector, then $\sum_j e^{i(\mathbf{k}'_s-\mathbf{k}_s+\mathbf{q}_s+\mathbf{g}_s)\cdot\mathbf{t}_j} = N_s\delta_{\mathbf{k}_s-\mathbf{k}'_s,\mathbf{q}_s+\mathbf{g}_s}$. Thus the interlayer Coulomb interaction becomes

$$H_{\text{gr-sub}} = \frac{1}{N_c}\sum_{\alpha,\sigma,\sigma'}\sum_{\mathbf{k},\mathbf{k}_s,\mathbf{q}_s,\mathbf{g}_s}\sum_{\widetilde{\mathbf{R}}} V(|\widetilde{\mathbf{R}} + d\hat{\mathbf{z}}|)\, e^{i(\mathbf{q}_s+\mathbf{g}_s)\cdot\widetilde{\mathbf{R}}}\, \hat{c}^{\dagger}_{\sigma\alpha}(\mathbf{k})\, \hat{d}^{\dagger}_{\sigma'}(\mathbf{k}_s)\, \hat{d}_{\sigma'}(\mathbf{k}_s - \mathbf{q}_s - \mathbf{g}_s)\, \hat{c}_{\sigma\alpha}(\mathbf{k} + \mathbf{q}_s + \mathbf{g}_s)\ , \tag{S73}$$

where $\widetilde{\mathbf{R}} = \mathbf{a}_i + \boldsymbol{\tau}_{\alpha} - \mathbf{t}_j$. Then,

$$\begin{aligned} &\frac{1}{N_c}\sum_{\widetilde{\mathbf{R}}} V(|\widetilde{\mathbf{R}} + d\hat{\mathbf{z}}|)\, e^{i(\mathbf{q}_s+\mathbf{g}_s)\cdot\widetilde{\mathbf{R}}} \\ =&\frac{1}{N_c\Omega_0}\sum_{\widetilde{\mathbf{R}}} \Omega_0\, V(|\widetilde{\mathbf{R}} + d\hat{\mathbf{z}}|)\, e^{i(\mathbf{q}_s+\mathbf{g}_s)\cdot\widetilde{\mathbf{R}}} \\ =&\frac{1}{S}\int d^2\mathbf{r}\, V(|\mathbf{r} + d\hat{\mathbf{z}}|)\, e^{i(\mathbf{q}_s+\mathbf{g}_s)\cdot\mathbf{r}} \\ =&\frac{e^2\, e^{-|\mathbf{q}_s+\mathbf{g}_s|\,d}}{2\epsilon_0\epsilon_r|\mathbf{q}_s + \mathbf{g}_s|\,S} \end{aligned} \tag{S74}$$

where $S = N_c\Omega_c$ is the total area of the system. Plugging Eq. (S74) into Eq. (S73), one obtains

$$\begin{aligned} H_{\text{gr-sub}} &= \frac{1}{S}\sum_{\alpha,\sigma,\sigma'}\sum_{\mathbf{k},\mathbf{k}_s,\mathbf{q}_s,\mathbf{g}_s} \frac{e^2\, e^{-|\mathbf{q}_s+\mathbf{g}_s|\,d}}{2\epsilon_0\epsilon_r|\mathbf{q}_s+\mathbf{g}_s|}\, \hat{c}^\dagger_{\sigma\alpha}(\mathbf{k})\, \hat{d}^\dagger_{\sigma'}(\mathbf{k}_s)\, \hat{d}_{\sigma'}(\mathbf{k}_s-\mathbf{q}_s-\mathbf{g}_s)\, \hat{c}_{\sigma\alpha}(\mathbf{k}+\mathbf{q}_s+\mathbf{g}_s) \\ &\approx \frac{1}{S}\sum_{\alpha,\sigma,\sigma'}\sum_{\mathbf{k},\mathbf{k}_s,\mathbf{q}_s} \frac{e^2\, e^{-|\mathbf{q}_s|\,d}}{2\epsilon_0\epsilon_r|\mathbf{q}_s|}\, \hat{c}^\dagger_{\sigma\alpha}(\mathbf{k})\, \hat{d}^\dagger_{\sigma'}(\mathbf{k}_s)\, \hat{d}_{\sigma'}(\mathbf{k}_s-\mathbf{q}_s)\, \hat{c}_{\sigma\alpha}(\mathbf{k}+\mathbf{q}_s) \\ &\approx \frac{1}{S}\sum_{\mu,\alpha,\sigma,\sigma'}\sum_{\mathbf{k},\mathbf{k}',\mathbf{q}} \frac{e^2\, e^{-|\mathbf{q}|\,d}}{2\epsilon_0\epsilon_r|\mathbf{q}|}\, \hat{c}^\dagger_{\sigma\mu\alpha}(\mathbf{k})\, \hat{d}^\dagger_{\sigma'}(\mathbf{k}')\, \hat{d}_{\sigma'}(\mathbf{k}'-\mathbf{q})\, \hat{c}_{\sigma\mu\alpha}(\mathbf{k}+\mathbf{q}) \end{aligned} \tag{S75}$$

where in the second line of the above equation we only keep the $\mathbf{g}_s = \mathbf{0}$ scattering channels due to the exponential decaying form of the interlayer Coulomb interactions in reciprocal space. In the last line of the above equation, we expand the wavevectors of electrons in the graphene layer around the Dirac point, i.e., let $\mathbf{k} \to \mathbf{K}_\mu + \mathbf{k}$, then assign valley index μ to the annihilation (creation) operators, $\hat{c}^{(\dagger)}_{\sigma\alpha}(\mathbf{k}) \to \hat{c}^{(\dagger)}_{\sigma\mu\alpha}(\mathbf{k})$. We have also neglected the intervalley Coulomb scattering for the electrons in the graphene layer arising from the interlayer Coulomb interactions, which is an excellent approximation given that the superlattice constant L_s is much larger than graphene's atomic lattice constant a. In the last line of the above equation, we have also let $\mathbf{k}_s \to \mathbf{k}'$, $\mathbf{q}_s \to \mathbf{q}$, in the sense that if we are interested in the low-energy states in both the graphene layer and the substrate layer, we do not have to distinguish whether the wavevector is defined in graphene's or substrate's Brillouin zone as those wavevectors are far from reaching the Brillouin zone boundary. Note that the mathematical derivations are for now practically the same as in Supplementary Note 1. However, The idea of using atomic lattice $\{\mathbf{t}_j\}$ in substrate would make a difference. In short, a charge-ordered state would emerge spontaneously while treating together the intralayer e-e Coulomb interactions Eq. (S67) and the kinetic part Eq. (S66). The spatial distribution of charges is localized, but it should also be smooth on the superlattice scale. We will show the technical details in the next subsection. After collecting all the contributions, we obtain the Hamiltonians Eqs. (5a-e) in the main text. Comparing with what we have studied in Supplementary Notes 1-5, the really new flavors we add into the menu are the kinetic energy and Coulomb interactions in the substrate. In the following, we provide technical details on: first, Hartree-Fock calculations on the substrate's side, which offers us charge modulation of the EC state; second, perturbative approach to estimate the stabilizing effect via interlayer Coulomb potential.

Hartree-Fock calculations on the coupled bilayer system

We first start working on the sum of the Hamiltonians Eq. (5b) and (5d). If electronic-crystal state is formed, electrons will be spontaneously arranged in a superlattice. This amounts to fold the plane wave wavevector $\mathbf{k}$ in the mini Brillouin zone of the presumed lattice so that $\mathbf{k} = \widetilde{\mathbf{k}} + \mathbf{G}$, where $\widetilde{\mathbf{k}}$ is within the first mini Brillouin zone and $\mathbf{G}$ is a reciprocal lattice vector of the superlattice. In Fig. 4 of the main text, we have considered the case of a triangular superlattice, which is the actual ground state of Wigner crystal for free 2D electron gas. This leads to

$$\hat{d}_{\sigma,\mathbf{G}}(\widetilde{\mathbf{k}}) \equiv \hat{d}_\sigma(\mathbf{k}) \tag{S76}$$

so that the kinetic part becomes

$$H^0_{\text{sub}} = \sum_{\widetilde{\mathbf{k}},\mathbf{G},\sigma} \left(\frac{\hbar^2(\widetilde{\mathbf{k}}+\mathbf{G})^2}{2m^*} + E_{\text{CBM}} \right) \hat{d}^\dagger_{\sigma,\mathbf{G}}(\widetilde{\mathbf{k}})\, \hat{d}_{\sigma,\mathbf{G}}(\widetilde{\mathbf{k}})\,. \tag{S77}$$

Similarly, we also write momentum transfer $\mathbf{q}$ in terms of $\widetilde{\mathbf{q}} + \mathbf{Q}$ so that Eq. (5d) becomes

$$H^{\text{intra}}_{\text{sub}} = \frac{1}{2S}\sum_{\widetilde{\mathbf{k}},\widetilde{\mathbf{k}}',\widetilde{\mathbf{q}}}\ \sum_{\substack{\sigma,\sigma' \\ \mathbf{G},\mathbf{G}',\mathbf{Q}}} V_{\text{int}}(\widetilde{\mathbf{q}}+\mathbf{Q})\, \hat{d}^\dagger_{\sigma,\mathbf{G}+\mathbf{Q}}(\widetilde{\mathbf{k}}+\widetilde{\mathbf{q}})\, \hat{d}^\dagger_{\sigma',\mathbf{G}'-\mathbf{Q}}(\widetilde{\mathbf{k}}'-\widetilde{\mathbf{q}})\, \hat{d}_{\sigma',\mathbf{G}'}(\widetilde{\mathbf{k}}')\, \hat{d}_{\sigma,\mathbf{G}}(\widetilde{\mathbf{k}})\,. \tag{S78}$$

where we always use a dielectric constant $\epsilon_r = 4$ in this section.

Then, we treat the interacting part Eq. (5b) with standard HF approximations, whose formalism exactly parallel with what we have done for graphene except simpler with only spin index. By momentum and spin conservation, where

$$\left\langle \hat{d}^\dagger_{\sigma,\mathbf{G}+\mathbf{Q}}(\widetilde{\mathbf{k}}+\widetilde{\mathbf{q}})\, \hat{d}_{\sigma',\mathbf{G}'}(\widetilde{\mathbf{k}}') \right\rangle_d = \left\langle \hat{d}^\dagger_{\sigma,\mathbf{G}+\mathbf{Q}}(\widetilde{\mathbf{k}}')\, \hat{d}_{\sigma,\mathbf{G}'}(\widetilde{\mathbf{k}}') \right\rangle_d \delta_{\widetilde{\mathbf{k}}+\widetilde{\mathbf{q}},\widetilde{\mathbf{k}}'}\delta_{\sigma,\sigma'} \tag{S79}$$

So, the Hartree term reads

$$V_{\rm sub}^{\rm H} = \frac{1}{S}\sum_{\widetilde{\mathbf{k}},\widetilde{\mathbf{k}}'}\sum_{\substack{\sigma,\sigma'\\ \mathbf{G},\mathbf{G}',\mathbf{Q}}} V_{\rm int}(\mathbf{Q})\,\left\langle \hat{d}^{\dagger}_{\sigma',\mathbf{G}'-\mathbf{Q}}(\widetilde{\mathbf{k}}')\hat{d}_{\sigma',\mathbf{G}'}(\widetilde{\mathbf{k}}')\right\rangle_d \hat{d}^{\dagger}_{\sigma',\mathbf{G}+\mathbf{Q}}(\widetilde{\mathbf{k}})\,\hat{d}_{\sigma,\mathbf{G}}(\widetilde{\mathbf{k}})\;. \tag{S80}$$

and the Fock term reads

$$\begin{aligned} V_{\rm sub}^{\rm F} &= -\frac{1}{S}\sum_{\widetilde{\mathbf{k}},\widetilde{\mathbf{k}}'}\sum_{\substack{\sigma,\sigma'\\ \mathbf{G},\mathbf{G}',\mathbf{Q}}} V_{\rm int}(\widetilde{\mathbf{k}}'-\widetilde{\mathbf{k}}+\mathbf{Q})\,\delta_{\sigma,\sigma'}\,\left\langle \hat{d}^{\dagger}_{\sigma,\mathbf{G}+\mathbf{Q}}(\widetilde{\mathbf{k}}')\hat{d}_{\sigma,\mathbf{G}'}(\widetilde{\mathbf{k}}')\right\rangle_d \hat{d}^{\dagger}_{\sigma,\mathbf{G}'-\mathbf{Q}}(\widetilde{\mathbf{k}})\,\hat{d}_{\sigma,\mathbf{G}}(\widetilde{\mathbf{k}}) \\ &= -\frac{1}{S}\sum_{\widetilde{\mathbf{k}},\widetilde{\mathbf{k}}'}\sum_{\substack{\sigma,\sigma'\\ \mathbf{G},\mathbf{G}',\mathbf{Q}}} V_{\rm int}(\widetilde{\mathbf{k}}'-\widetilde{\mathbf{k}}+\mathbf{Q}+\mathbf{G}-\mathbf{G}')\,\delta_{\sigma,\sigma'}\,\left\langle \hat{d}^{\dagger}_{\sigma,\mathbf{G}'-\mathbf{Q}}(\widetilde{\mathbf{k}}')\hat{d}_{\sigma,\mathbf{G}'}(\widetilde{\mathbf{k}}')\right\rangle_d \hat{d}^{\dagger}_{\sigma,\mathbf{G}+\mathbf{Q}}(\widetilde{\mathbf{k}})\,\hat{d}_{\sigma,\mathbf{G}}(\widetilde{\mathbf{k}}) \end{aligned} \tag{S81}$$

where we recenter $\mathbf{Q}$ to get the last line. Here, $\langle\ldots\rangle_d$ means the expectation value of observable after integrating only the substrate part of the many-body ground state $|\Psi\rangle_d^0$. Spin polarization is allowed in the Hartree-Fock treatment of the 2D electron gas in the substrate, and indeed a spin polarized Wigner crystal state always has a lower energy than a spin degenerate one.

The HF calculations have been done for a series of different superlattice constant L_s. Once L_s is given, the charge density in substrate is then fixed by $n_d = g_v/(\sqrt{3}L_s^2/2)$ for a spin polarized Wigner crystal state with triangular superlattice, where $g_v = 2$ comes from the valley degeneracy of the conduction band minimum of CrOCl. Here we set up our parameters including the effective mass $m^* = 1.3m_0$, the background dielectric constant $\epsilon_r = 4$, and valley degeneracy $g_v = 2$, to mimic the properties of conduction band minimum of CrOCl. Such mapping amounts to always let only the lowest subband in the folded Brillouin zone be filled. During the iterations, we keep track all the subbands. To initialize the HF self-consistent loop, the terms like $\left\langle \hat{d}^{\dagger}_{\sigma,\mathbf{G}'-\mathbf{Q}}(\widetilde{\mathbf{k}}')\hat{d}_{\sigma,\mathbf{G}'}(\widetilde{\mathbf{k}}')\right\rangle_d$ are set to be non-zero, corresponding to a spontaneous charge order with Fourier component $\mathbf{Q}$, where $\mathbf{Q}$ is one of the primitive reciprocal vectors for the triangular superlattice. In other words, we start the HF loop from a charge-ordered state, which facilitates the convergence to the same phase. If the final converged state is gapped, we can extract its charge modulation as input for the next step. In our calculations, a 9×9 mesh of reciprocal lattice (centered at Γ point) has been adopted, with the mini Brillouin zone sampled by a 18×18 $\mathbf{k}$ mesh. The former corresponds to the real-space mesh within a primitive cell, while the latter corresponds to the system size. We have also performed calculations adopting a 13×13 mesh of reciprocal lattice points (corresponding to finer real-space mesh), and find completely consistent results with those calculated using a 9×9 reciprocal lattice points.

With the HF results on the substrate side in hands, we are ready to study the rest of three Hamiltonians, namely Eqs. (5a), (5c) and (5e) of the main text. The mean-field treatment is exactly the same as in Supplementary Notes 3 and 4. However, the interlayer coupling Hamiltonian Eq. (5e) worth special attention. Under the separable wavefunction ansatz, we can still integrate out all the d-operators using the wavefunctions resulted from the previous HF calculations solely on the substrate side:

$$\begin{aligned} \langle H_{\text{gr-sub}}\rangle_d =& \frac{1}{S}\sum_{\mu,\alpha,\sigma,\sigma'}\sum_{\mathbf{k},\mathbf{k}',\mathbf{q}} \frac{e^2\,e^{-|\mathbf{q}|\,d}}{2\epsilon_0\epsilon_r|\mathbf{q}|}\,\hat{c}^{\dagger}_{\sigma\mu\alpha}(\mathbf{k})\,\left\langle \hat{d}^{\dagger}_{\sigma'}(\mathbf{k}')\,\hat{d}_{\sigma'}(\mathbf{k}'-\mathbf{q})\right\rangle_d \hat{c}_{\sigma\mu\alpha}(\mathbf{k}+\mathbf{q}) \\ =& \frac{1}{S}\sum_{\mu,\alpha,\sigma,\sigma'}\sum_{\widetilde{\mathbf{k}},\widetilde{\mathbf{k}}',\widetilde{\mathbf{q}}}\sum_{\mathbf{G},\mathbf{G}',\mathbf{Q}} \frac{e^2\,e^{-|\widetilde{\mathbf{q}}+\mathbf{Q}|\,d}}{2\epsilon_0\epsilon_r|\widetilde{\mathbf{q}}+\mathbf{Q}|}\,\hat{c}^{\dagger}_{\sigma\mu\alpha}(\widetilde{\mathbf{k}}+\mathbf{G})\,\left\langle \hat{d}^{\dagger}_{\sigma',\mathbf{G}'}(\widetilde{\mathbf{k}}')\,\hat{d}_{\sigma',\mathbf{G}'-\mathbf{Q}}(\widetilde{\mathbf{k}}'\widetilde{\mathbf{q}})\right\rangle_d \hat{c}_{\sigma\mu\alpha}(\widetilde{\mathbf{k}}+\mathbf{G}+\widetilde{\mathbf{q}}+\mathbf{Q}) \\ =& \frac{1}{S}\sum_{\mu,\alpha,\sigma,\sigma'}\sum_{\widetilde{\mathbf{k}},\widetilde{\mathbf{k}}'}\sum_{\mathbf{G},\mathbf{G}',\mathbf{Q}} \frac{e^2\,e^{-|\mathbf{Q}|\,d}}{2\epsilon_0\epsilon_r|\mathbf{Q}|}\,\hat{c}^{\dagger}_{\sigma\mu\alpha,\mathbf{G}}(\widetilde{\mathbf{k}})\,\left\langle \hat{d}^{\dagger}_{\sigma',\mathbf{G}'}(\widetilde{\mathbf{k}}')\,\hat{d}_{\sigma',\mathbf{G}'-\mathbf{Q}}(\widetilde{\mathbf{k}}')\right\rangle_d \hat{c}_{\sigma\mu\alpha,\mathbf{G}+\mathbf{Q}}(\widetilde{\mathbf{k}}) \\ =& \sum_{\sigma,\mu,\alpha}\sum_{\widetilde{\mathbf{k}},\mathbf{G},\mathbf{Q}} \frac{e^2\,e^{-|\mathbf{Q}|\,d}}{2\epsilon_0\epsilon_r\,\Omega_d\,|\mathbf{Q}|}\,\rho_d(\mathbf{Q})\,\hat{c}^{\dagger}_{\sigma\mu\alpha,\mathbf{G}}(\widetilde{\mathbf{k}})\,\hat{c}_{\sigma\mu\alpha,\mathbf{G}+\mathbf{Q}}(\widetilde{\mathbf{k}})\;. \end{aligned} \tag{S82}$$

Here, we define the charge modulation $\rho_d(\mathbf{Q})$ of the charge-ordered state as

$$\begin{aligned}\rho_d(\mathbf{Q}) &= \frac{1}{N_d}\sum_{\widetilde{\mathbf{k}}',\mathbf{G}',\sigma'}\left\langle \hat{d}^\dagger_{\sigma',\mathbf{G}'}(\widetilde{\mathbf{k}}')\,\hat{d}_{\sigma',\mathbf{G}'-\mathbf{Q}}(\widetilde{\mathbf{k}}')\right\rangle_d \\ &= \frac{1}{N_d}\sum_{\widetilde{\mathbf{k}}',\mathbf{G}',\sigma'}\sum_n D^*_{\sigma\mathbf{G}'+\mathbf{Q},n}(\widetilde{\mathbf{k}}')\,D_{\sigma\mathbf{G}',n}(\widetilde{\mathbf{k}}')\,\theta(E_F - E^d_{n\widetilde{\mathbf{k}}'})\,,\end{aligned} \tag{S83}$$

where we write the expectation values in the substrate's subband basis in the presence of HF potentials

$$\hat{d}_{\sigma,\mathbf{G}}(\widetilde{\mathbf{k}}) = \sum_n D_{\sigma\mathbf{G},n}(\widetilde{\mathbf{k}})\,\hat{d}_{\sigma,n}(\widetilde{\mathbf{k}})\,. \tag{S84}$$

$\{D_{\sigma\mathbf{G},n}(\widetilde{\mathbf{k}})\}$ relates precisely the d-operators in the original plane-wave basis to that in the Bloch-function basis, and $E^d_{n\widetilde{\mathbf{k}}'}$ denotes the subband energy dispersion. Remarkably, the two conceptually different treatments give rise to an interlayer Coulomb potential with the same analytical properties. It justifies our previous drastic assumptions on the localization of charge distribution. Here we only do better since we can self-consistently solve the charge-ordered ground state of the substrate and its charge density distributions.

Exactly parallel with what we have done earlier, we now solve the problem of graphene on the superlattice defined $\rho_d(\mathbf{Q})$ while only keeping the dominant terms with small $\mathbf{Q}$. The e-e interactions are still treated in the band basis by HF approximations in a low-energy window given by the band index cut-off. We keep track only three valence and three conduction bands per valley per spin. The contributions from high-energy bands are taken into account using the RG approach, replacing the Fermi velocity by a renormalized one. In the continuum model treatment, a 9×9 mesh of reciprocal lattice points has been adopted, and the mini Brillouin zone is sampled in a 18×18 $\mathbf{k}$ mesh. The filling of graphene is set to be at the CNP so that a sublattice gap would be opened in the presence of superlattice potential. Once found the final converged ground state using all the types of initial order parameters, we calculate the charge modulation of gapped graphene $\rho_c(\mathbf{Q})$

$$\rho_c(\mathbf{Q}) = \frac{1}{N_c}\sum_{\sigma,\mu,\alpha}\sum_{\widetilde{\mathbf{k}},\mathbf{G}}\left\langle \hat{c}^\dagger_{\sigma\mu\alpha,\mathbf{G}}(\widetilde{\mathbf{k}})\,\hat{c}_{\sigma\mu\alpha,\mathbf{G}-\mathbf{Q}}(\widetilde{\mathbf{k}})\right\rangle_c \tag{S85}$$

where $\langle\ldots\rangle_c$ is the expectation value of integrating only the graphene part of the many-body ground state $|\Psi\rangle^0_c$.

So far, we have separately found the HF ground state for substrate only and that for graphene coupled to the superlattice potential arising from the charge-ordered state in substrate. We note $\langle\ldots\rangle_{\text{HF}}$ as the expectation value of observable using the HF ground state, i.e., charge-ordered state for substrate and gapped state for graphene. As a reference, we also note $\langle\ldots\rangle_{\text{FL}}$ as the expectation value of observable using the non-interacting plane-wave state, i.e., 2D electron gas for substrate and non-interacting Dirac fermions for graphene. If the charge-ordered state cooperates with the gapped state in graphene, the coupled bilayer system must be energetically more stable than the substrate alone. Explicitly, this means that the condensation energy $E_{\text{cond, sub}} > E_{\text{cond, coupled}}$, where

$$E_{\text{cond, sub}} = \langle H^0_{\text{sub}} + H^{\text{intra}}_{\text{sub}}\rangle_{\text{HF}} - \langle H^0_{\text{sub}} + H^{\text{intra}}_{\text{sub}}\rangle_{\text{FL}} \tag{S86}$$

$$E_{\text{cond, coupled}} = \langle H^0_{\text{sub}} + H^{\text{intra}}_{\text{sub}} + H^0_{\text{gr}} + H^{\text{intra}}_{\text{gr}}\rangle_{\text{HF}} - \langle H^0_{\text{sub}} + H^{\text{intra}}_{\text{sub}} + H^0_{\text{gr}} + H^{\text{intra}}_{\text{gr}}\rangle_{\text{FL}} + E_{\text{gr-sub,opt}}\,. \tag{S87}$$

$E_{\text{gr-sub,opt}}$ is the interlayer Coulomb energy resulted from non-uniform charge modulations of the two layers, after optimization with respect to relative charge-center shift. It will be treated using perturbation theory shortly.

Besides, there are two subtleties in this comparison. First one is related to the cut-off. When we study graphene in the presence of superlattice potential, only the bands in low-energy window are considered. In principle, we need to add the energy contribution of all the electrons in the occupied bands. However, this would not make a significant difference since electrons deep in the valence bands are little affected by all the interaction effects discussed here. Particularly, the relevant interaction energy scale considered in this work is always far below our choice of low-energy cutoff for graphene ($\sim 3\times\hbar v_F 2\pi/L_s$). On the other hand, the contribution from the remote energy bands outside the low-energy window has already been included in our RG approach. Our second concern comes from the feedback effect from graphene to substrate. In principle, the non-uniform charge modulation of graphene would also affect the profile of the charge density distribution of the electronic-crystal state in the substrate by minimizing the interlayer Coulomb interaction. However, such interlayer Coulomb potential is weak compared to the intralayer counterpart for the substrate, as shown in Supplementary Figure 12. This allows us to treat the feedback effect perturbatively, i.e., include $\langle H_{\text{gr-sub}}\rangle_c$ as a perturbation to $H^0_{\text{sub}} + H^{\text{intra}}_{\text{sub}}$.

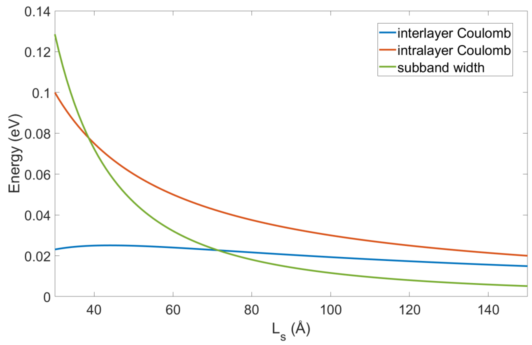

Supplementary Figure 12: Order of magnitudes of subband width (green) and intra- (red) and inter-layer (blue) Coulomb potential strength for different L_s. The dielectric constant is selected to be 4.

The first order effect is precisely the charge interlocking between graphene and substrate. There exists an optimal relative shift vector $\mathbf{t}_m$ between the two layers that minimize the interlayer Coulomb energy cost

$$E^{(1)}_{\text{gr-sub}} = N_c \sum_{\mathbf{Q}\neq\mathbf{0}} \frac{e^2\, e^{-|\mathbf{Q}|\,d}}{2\epsilon_0\epsilon_r\,\Omega_d\,|\mathbf{Q}|}\, e^{-i\mathbf{Q}\cdot\mathbf{t}_m}\, \rho_d(\mathbf{Q})\,\rho_c(-\mathbf{Q})\;. \tag{S88}$$

However, to the second order, one has to consider how the HF wavefunction of the electronic-crystal state in substrate is changed by the charge modulation of graphene. In the HF subband basis,

$$\begin{aligned}\langle H_{\text{gr-sub}}\rangle_c &= \sum_{\mathbf{Q}\neq\mathbf{0}} \sum_{\widetilde{\mathbf{k}},\mathbf{G},\sigma} \frac{e^2\, e^{-|\mathbf{Q}|\,d}}{2\epsilon_0\epsilon_r\,\Omega_d\,|\mathbf{Q}|}\,\rho_c(-\mathbf{Q})\,\hat{d}^\dagger_{\sigma,\mathbf{G}}(\widetilde{\mathbf{k}})\,\hat{c}_{\sigma,\mathbf{G}-\mathbf{Q}}(\widetilde{\mathbf{k}}) \\ &= \sum_{\mathbf{Q}\neq\mathbf{0}} \sum_{\widetilde{\mathbf{k}},\mathbf{G},\sigma} \frac{e^2\, e^{-|\mathbf{Q}|\,d}}{2\epsilon_0\epsilon_r\,\Omega_d\,|\mathbf{Q}|}\,\rho_c(-\mathbf{Q}) \sum_{n,m} D^*_{\sigma\mathbf{G}'+\mathbf{Q},m}(\widetilde{\mathbf{k}})\,D_{\sigma\mathbf{G}',n}(\widetilde{\mathbf{k}})\hat{d}^\dagger_{\sigma,m}(\widetilde{\mathbf{k}})\,\hat{d}_{\sigma,n}(\widetilde{\mathbf{k}})\,.\end{aligned} \tag{S89}$$

The energy shift due to such modification is

$$E^{(2)}_{\text{gr-sub}} = \sum_{\widetilde{\mathbf{k}},\sigma} \sum_{\substack{n\in\text{occ.}\\ m\in\text{emp.}}} \frac{\left|\sum_{\mathbf{G}',\mathbf{Q}} \frac{e^2\, e^{-|\mathbf{Q}|\,d}}{2\epsilon_0\epsilon_r\,\Omega_d\,|\mathbf{Q}|}\, e^{-i\mathbf{Q}\cdot\mathbf{t}_m}\,\rho_c(-\mathbf{Q})\,D^*_{\sigma\mathbf{G}'+\mathbf{Q},m}(\widetilde{\mathbf{k}})\,D_{\sigma\mathbf{G}',n}(\widetilde{\mathbf{k}})\right|^2}{E^d_{n\widetilde{\mathbf{k}}} - E^d_{m\widetilde{\mathbf{k}}}} < 0\,, \tag{S90}$$

where $\mathbf{Q}\neq\mathbf{0}$ in the above equation. Note that $E^{(2)}_{\text{gr-sub}}$ is also sensitive to the relative shift between the two layers so one need to find the optimal $\mathbf{t}_{\text{opt}}$ such that it minimizes the sum of $E^{(1)}_{\text{gr-sub}}$ and $E^{(2)}_{\text{gr-sub}}$, namely $E_{\text{gr-sub,opt}}$ in Eq. (S87). This is the value we need to use in the comparison between two condensation energy $E_{\text{cond, sub}}$ and $E_{\text{cond, coupled}}$. We also note that all the kinetic energies and intralayer Coulomb interaction energies are unchanged under the relative shift between the two layers. The results will answer the question on how synergistic correlated states occur in such coupled bilayer heterostructure.

SUPPLEMENTARY NOTE 7: DETAILS OF DFT CALCULATIONS FOR THE SUBSTRATE MATERIALS

Lattice structures, deformation potentials, and band structures of candidate substrate materials

In this section and the following one, we present the details for the density function theory (DFT) calculations of the 14 candidate substrate materials presented in Table 1 of the main text. In this subsection, we show the results for all but CrOCl and transition dichalcogenides. These materials, which worth special attention, are given in the next subsections. The first principles calculations are performed with the projector augmented-wave method within the density functional theory [10], as implemented in the Vienna ab initio simulation package software [11]. The crystal

structure is fully optimized until the energy difference between two successive steps is smaller than 10^{-6} eV and the Hellmann-Feynman force on each atom is less than 0.01 eV/Å. The generalized gradient approximation by Perdew, Burke, and Ernzerhof is taken as the exchange–correlation potential [12]. As Cr is a transition metal element with localized $3d$ orbitals, we use the on-site Hubbard parameter $U = 5.48$ eV for the Cr $3d$ orbitals in the CrOCl bilayer and $U = 3$ eV for Cr $3d$ orbitals in the CrI_3 bilayer. The so-called fully localized limit of the spin-polarized GGA+U functional is adopted as suggested by Liechtenstein and coworkers [13], and the non-spherical contributions from the gradient corrections are taken into consideration. The "DFT+D2" type of vdW correction has been adopted for all multilayer calculations to properly describe the interlayer interactions [14].

Our high-throughput filtering of the proper insulating substrate materials for graphene starts from the 2D materials computational database [15]. We only focus at those with bulk van der Waals structures which have been previously synthesized in laboratory. This ensures that it is experimentally feasible to exfoliate few layers from their bulk sample and then stack them on graphene to form heterostructures. The results are summarized in Table 1 of the main text.

The lattice structures of some of the substrate materials presented in Table 1 of the main text are shown in Supplementary Figure 13. They are either in the bilayer or trilayer structures. The lattice structure of CrI_3 is similar to that of YI_3 as shown in Supplementary Figure 13(d). Their band structures are presented in Supplementary Figure 14, where the green dashed lines mark the energy position of the Dirac point in graphene. We note that the valence band maximum (VBM) of PbO bilayer is energetically close to the Dirac point of graphene; while for the other bilayer or trilayer substrate materials, their conduction band minima (CBM) are close to the Dirac point. This indicates that charge transfer can easily occur between graphene and the substrates controlled by gate voltages. Moreover, we note that the conduction bands and valence bands of these materials are typically flat with large effective masses, which would be very susceptible to e-e Coulomb interactions once these substrate materials are slightly charge doped, and may lead to Wigner-crystal-like state or long-wavelength ordered state as discussed in main text. Another important precondition for the Wigner-crystal state is that the screening effect of substrate materials can not be too strong. For example, the conduction band of ScOBr bilayer has a large effective mass of $2.575m_0$ (m_0 is the bare mass of a free electron), but the dielectric constant ϵ_r of ScOBr reaches $\sim$13, which makes it difficult to trigger the Wigner-crystal-like instability in this material under slight charge doping.

We note that all of these proposed substrate materials all have been successfully synthesized in laboratory as listed in Supplementary Table 4. Especially, few-layer of $ReSe_2$ as a highly anisotropic material [16–18], and few-layer CrI_3 system as a 2D magnetic material [19–22], have been extensively studied recently. Moreover, phonon spectra calculations have proved the dynamical stability of these substrate materials in monolayer from [15]. Thus the device fabrication of heterostructure consisting of graphene monolayer and one of these candidate substrate materials should be experimentally accessible.

There always exists tension or compression in a heterostructure system. Under some lattice deformation, the variation of conduction band minimum (CBM) or valence band maximum (VBM) is defined as deformation potential. We list the deformation potentials of the candidate substrate materials in Supplementary Table 4. We note that the maximum value of the deformation potential is only 5.84 eV for ScOCl, which means that the energy level of CBM of ScOCl would move down by only 0.063 eV under 1% tensile strain. Therefore, even if strain is introduced in the graphene-insulator heterostructure proposed in this work, the band edges (with large effective masses) of those candidate substrate materials are still energetically close to the Dirac point of graphene.

In these candidate materials (except for CrOCl), CrI_3 bilayer is the only magnetic system. Previous theoretical studies reveal that the stacking configuration of CrI_3 bilayer plays an important role in the magnetic ground state [23]. Here we use the AB′-type stacking in the bilayer structure, which is consistent with the stacking configuration in the bulk phase of CrI_3. The AB′-stacked CrI_3 bilayer is in an intralayer ferromagnetic and interlayer antiferromagnetic ground state.

Band structure of graphene-CrOCl heterostructure

We study graphene-CrOCl heterostructure using DFT calculations to show the small orbital overlap between graphene's carbon atom and CrOCl's Cr atom. Technically, we need to construct a commensurate supercell matching graphene and CrOCl's primitive unit-cell.

In our modeling, we use an $8 \times \sqrt{7}$ supercell of graphene. We refer $\mathbf{a}_1$ and $\mathbf{a}_2$ to the lattice vectors along the short and long axis, respectively. Then, the corresponding supercell for CrOCl is a $(2\mathbf{a}_1, \mathbf{a}_1 + 5\mathbf{a}_2)$ one. The average mismatch is $\sim 0.7\%$. In particular, there are 0.4% tensile stress along long axis and 2.0% compressive strain along short axis in the graphene supercell.

The band structure is shown in Supplementary Figure 16. The lattice distortion amounts to adding strain to

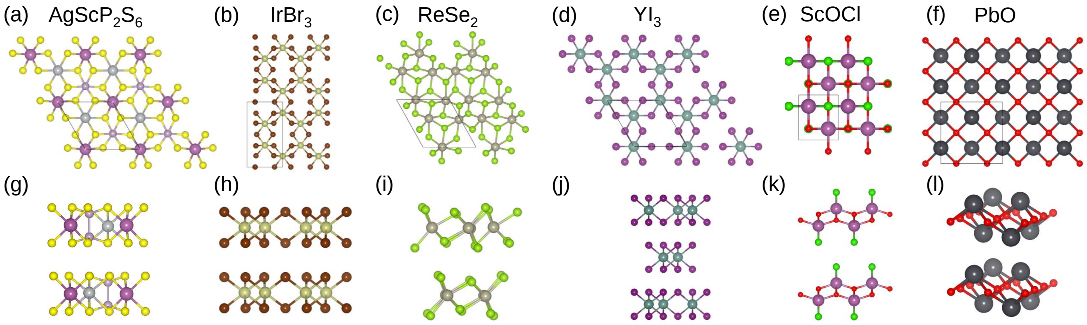

Supplementary Figure 13: (a)-(f): top views of the lattice structures of some candidate substrate materials in monolayer form. The primitive cells are remarked with black lines. (g)-(l): the side views of these substrate materials in few-layer form.

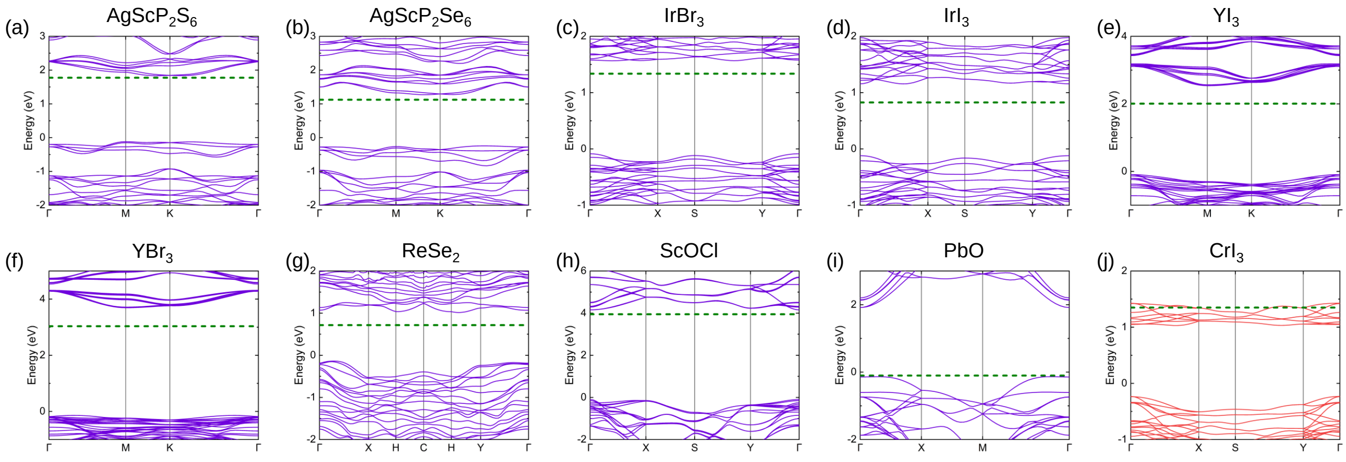

Supplementary Figure 14: The calculated energy bands of the candidate substrate materials, where the energy position of the Dirac point of graphene is marked by a dashed green line in the band structures. We single out CrI_3 by plotting its band structure using red solid lines. This is because CrI_3 is the only magnetic system among these ten materials so that it worth special attention on the magnetic nature of its ground state. Here, we suppose CrI_3 to have an intralayer ferromagnetic and interlayer antiferromagnetic ground state.

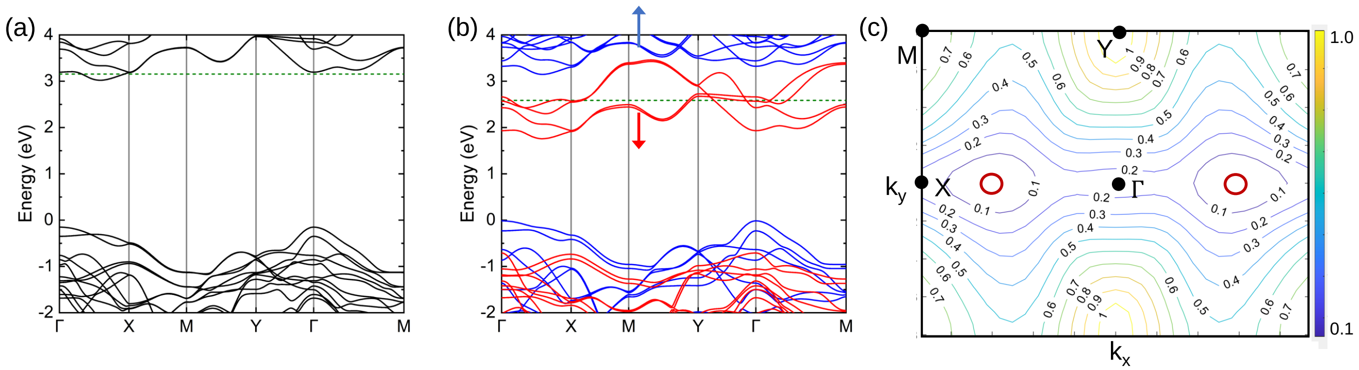

Supplementary Figure 15: The calculated energy bands of antiferromagnetic bilayer CrOCl: (a) without electric field, and (b) with an electric field 0.3 V/nm. In (b), the energy bands from top and bottom layers are marked by red and blue lines, respectively. The energy position of the Dirac point of graphene are remarked with green dashed lines. (c) Fermi surface of bilayer CrOCl at different Fermi levels with respect to the conduction band minimum. The Fermi surface under 1/100 electron filling factor is remarked by red circles.

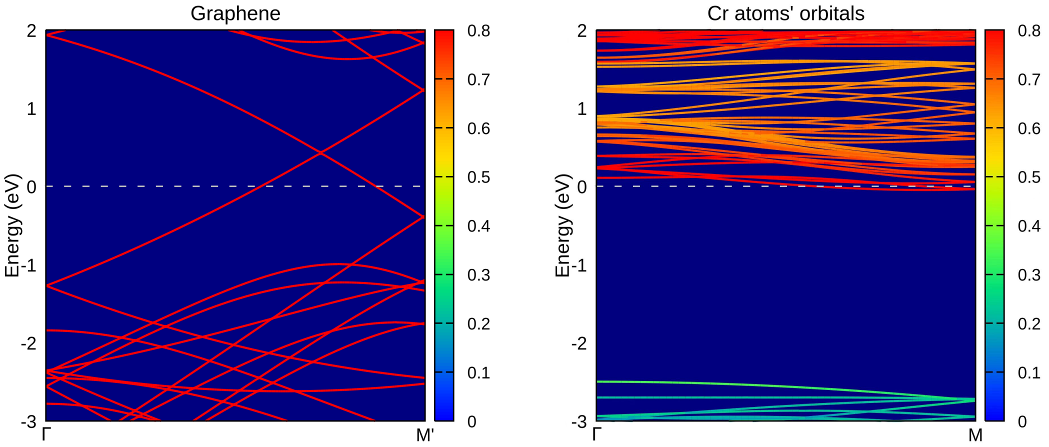

Supplementary Figure 16: Graphene-CrOCl heterostructure's non-interacting bands, in which we project the orbital contribution on the two sides of the heterostructure: graphene's C atoms (left) and CrOCl's Cr atoms (right).

graphene and thus moves the position of Dirac cone away from high-symmetry lines. Therefore, we choose the line (from Γ to M', which is not a high-symmetry point but near M) that passes the Dirac point in the Brillouin zone to plot the band structure. We see that the orbital overlap is small and thus negligible.

Electric-field tunable band structures of bilayer CrOCl

Now we discuss the electronic structure of CrOCl bilayer under vertical electrical fields. Here we consider an intralayer ferromagnetic and interlayer antiferromagnetic state for the bilayer configuration, which turns out to be one of competing low-energy magnetic states, and is the magnetic ground state when the on-site Hubbard U value for the Cr $3d$ orbitals is large. [24] The calculated band gap of CrOCl bilayer with the DFT+U calculation is $3.13\,\mathrm{eV}$, which is close to that of HSE06 calculation ($3.12\,\mathrm{eV}$) [15]. The band structure of antiferromagnetic CrOCl bilayer is shown in Supplementary Figure 15(a), where the green dashed line marks the energy position of the Dirac point of graphene. Without vertical electric field, the Dirac point is slightly above the CBM of bilayer CrOCl. Applying a vertical electric field of $0.03\,\mathrm{V/nm}$ would push down the CBM as shown in Supplementary Figure 15(b). A closer inspection reveals that the top-layer conduction state (red lines) is pushed downwards while the bottom-layer state (blue lines) is pushed upward in energy as shown in Supplementary Figure 15(b), such that electron carriers in the graphene layer (if there is any) would be transferred to the top layer of CrOCl substrate, forming a Wigner-crystal-like state at the surface of CrOCl substrate given that the Wigner-Seitz radius of the CBM $\sim 55.7{-}74.2$ (with a relative dielectric constant $\epsilon_r = 3{-}4$) is above the threshold value ~ 31 (see Table 1 in the main text). Thus, our conjecture is supported by detailed first principles DFT calculations.

In Supplementary Figure 15(c) we also present the Fermi surfaces at different Fermi energies above the CBM of bilayer CrOCl. At very low carrier densities with small Fermi energy (CBM is set to zero), the Fermi surface consists of two nearly isotropic circles. For example, at filling factor 1/100 (corresponding to a carrier density $\sim 8\times10^{12}\,\mathrm{cm}^{-2}$), the Fermi surface is marked by the red circles. Such isotropic Fermi surface with large effective mass ($\sim 1.308m_0$) is likely to give rise to Wigner-crystal state as discussed in the main text. As the Fermi level further increases, the Fermi surfaces become more and more anisotropic.

Transition metal dichalcogenides as substrates for graphene

Now we focus on the few layers of transition metal dichalcogenides (TMD) which have attracted remarkable interest recently. Here we use the dielectric constants of the corresponding bulk TMD materials to estimate the condition for the onset of Wigner crystal states in these few-layer systems. We find that the conduction band minima of MoX_2 (X = S, Se, Te), WY_2 and PtY_2 (Y = S, Se) few-layer systems are close to the Dirac point of graphene, as shown in the Supplementary Table 5. Especially, the conduction band edges in the MoX_2 and WY_2 systems mainly originate from

Supplementary Table 4: The experimental works about the ten substrate materials, and the uni-axial deformation potentials of these materials [15].

Materials	References	Deformation potentials
$AgScP_2S_6$	Ref. [25]	–
$AgScP_2Se_6$	Ref. [26]	–
$IrBr_3$	Ref. [27]	-3.76 eV
IrI_3	Ref. [28]	-2.17 eV
YI_3	Ref. [29]	1.47 eV
YBr_3	Ref. [30]	1.43 eV
$ReSe_2$	Ref. [31]	-4.45 eV
ScOCl	Ref. [32]	-5.84 eV
PbO	Ref. [33]	-4.60 eV
CrI_3	Ref. [19–22]	-2.20 eV

Supplementary Table 5: Transition-metal dichalcogenides as substrate materials for graphene. The dielectric constants ϵ_r [34–36], conduction band minimum (CBM) position (E_{CBM}), the corresponding effective mass m^* at the CBM that is energetically close to the Dirac point, and the required critical doping concentration n_c to realize the Wigner crystal state (with Wigner-Seitz radii $r_s = g_v m^*/\sqrt{\pi n_c}\epsilon_r m_0 a_{\mathrm{B}} = 31$ [37]) are tabulated. For clarity, the energy level of the Dirac point in graphene is set to zero. "mono", "bi", "tri" and "quad" stand for monolayer, bilayer, trilayer and quadruple-layer configurations, respectively.

Materials	ϵ_r	E_{CBM}	m^*	g_v	n_c
MoS_2 (mono)	5.69	-0.06 eV	0.425 m_0	2	2.84×10^{11} cm^{-2}
MoS_2 (bi)	5.69	-0.06 eV	0.446 m_0	2	3.10×10^{11} cm^{-2}
MoS_2 (tri)	5.69	-0.06 eV	0.467 m_0	2	3.40×10^{11} cm^{-2}
MoS_2 (quad)	5.69	-0.25 eV	0.484 m_0	2	3.64×10^{11} cm^{-2}
$MoSe_2$ (mono)	7.29	0.36 eV	0.492 m_0	2	2.30×10^{11} cm^{-2}
$MoSe_2$ (bi)	7.29	0.31 eV	0.773 m_0	6	$5.10\ \times10^{12}$ cm^{-2}
$MoSe_2$ (tri)	7.29	0.07 eV	0.739 m_0	6	$4.65\ \times10^{12}$ cm^{-2}
$MoSe_2$ (quad)	7.29	-0.01 eV	0.730 m_0	6	$4.56\ \times10^{12}$ cm^{-2}
$MoTe_2$ (mono)	6.75	0.53 eV	0.471 m_0	2	2.46×10^{11} cm^{-2}
$MoTe_2$ (bi)	6.75	0.42 eV	0.749 m_0	6	5.58×10^{12} cm^{-2}
$MoTe_2$ (tri)	6.75	0.34 eV	0.711 m_0	6	5.04×10^{12} cm^{-2}
$MoTe_2$ (quad)	6.75	0.31 eV	0.701 m_0	6	4.89×10^{12} cm^{-2}
WS_2 (mono)	3.63	0.27 eV	0.468 m_0	2	8.38×10^{11} cm^{-2}
WS_2 (bi)	3.63	0.25 eV	0.477 m_0	2	8.60×10^{11} cm^{-2}
WS_2 (tri)	3.63	0.08 eV	1.155 m_0	6	4.59×10^{13} cm^{-2}
WS_2 (quad)	3.63	0.00 eV	1.146 m_0	6	4.53×10^{13} cm^{-2}
WSe_2 (mono)	4.07	0.53 eV	0.456 m_0	2	6.32×10^{11} cm^{-2}
WSe_2 (bi)	4.07	0.52 eV	0.479 m_0	2	6.98×10^{11} cm^{-2}
WSe_2 (tri)	4.07	0.47 eV	0.539 m_0	6	7.95×10^{12} cm^{-2}
WSe_2 (quad)	4.07	0.27 eV	0.532 m_0	6	7.74×10^{12} cm^{-2}
PtS_2 (mono)	12.34	-0.29 eV	0.418 m_0	6	5.19×10^{11} cm^{-2}
PtS_2 (bi)	12.34	-0.51 eV	0.601 m_0	6	1.08×10^{12} cm^{-2}
$PtSe_2$ (mono)	24.70	-0.03 eV	0.327 m_0	6	7.95×10^{10} cm^{-2}
$PtSe_2$ (bi)	24.70	-0.31 eV	0.412 m_0	6	1.26×10^{11} cm^{-2}

the transition metal Mo or W d orbitals, which are localized and may have large effective masses. If the conduction bands of these TMD systems are slightly carrier doped, the systems may form Wigner crystal states as long as their Wigner-Seitz radii $r_s > 31$. We list the critical carrier concentration to realize the Wigner crystal states in various TMD few layers in the Supplementary Table 5, and their energy bands are shown in Supplementary Figure 17. The conduction band minima in the Pt-based TMD systems are energetically close to the Dirac point of graphene, but the strong screening effect (large dielectric constants) may prevent the electrons to form Wigner crystal states in the Pt-based TMD system. The out-of-plane electric field can easily tune the electronic structure in the MoX_2 and WY_2 systems. A previous theoretical work indicated that an electric field of 0.3 V/nm can decrease the band gap by 0.2 eV [38]. Because of the excellent fabrication technology of the TMD system and their CBM close to the Dirac point, we believe that the graphene/Mo(W)-based TMD heterostructure can provide a feasible platform to realize gapped

Dirac state concomitant with interaction-enhanced Fermi velocities.

SUPPLEMENTARY NOTE 8: EXPERIMENTAL MEASUREMENTS OF THE GAPS IN GRAPHENE-CROCL HETEROSTRUCTURE

To test our theory of the band reconstruction of Dirac fermions in graphene coupled with a long-wavelength charge order, we considered a few candidate substrates, among which CrOCl is suitable for device fabrication because of its high air-stability and easy-exfoliatable nature. By designing a dual-gated structure, we used few-layered CrOCl as an bottom dielectric while few-layered hexagonal boron nitride (h-BN) was served as top gate dielectric. The top and bottom gate voltages can then be converted into doping and displacement fields for further data analysis. In this section, we include below detailed experimental data on the quality of sample, the device configuration, the measurement setup as well as how we do the thermal gap measurements.

Quality of sample, device configuration and measurement setup

The devices are made of graphene, h-BN, and CrOCl flakes, which are mechanically exfoliated from high quality bulk crystals. The vertical assembly of few-layered hBN, monolayer graphene and few-layered CrOCl were made using the polymer-assisted dry-transfer method. Electron beam lithography was done using a Zeiss Sigma 300 SEM with a Raith Elphy Quantum graphic writer. Top and bottom gates as well as contacting electrodes were fabricated with an e-beam evaporator, with typical thicknesses of Ti/Au $\sim 5/50$ nm. A cartoon illustration of the tested device is shown in Supplementary Figure 18(a), which includes h-BN/Graphene/CrOCl van der Waals heterostructure equipped with a top gate and a bottom gate. Supplementary Figure 18(b) and (c) show the $5\times$ magnification and $100\times$ magnification optical pictures of the device, respectively. There are no visible bubbles or wrinkles on the heterostructure. To ensure the cleanliness of the sample, we will use an AFM (atomic force microscope) tip to clean the Au bottom gate in a Contact mode before landing heterostructures onto these local metallic gates. A significant amount of PMMA can be removed via such a process to make sure the homogeneity of the gate electrical fields (Supplementary Figure 18(d,e)). The device studied in this work is the same device S40 we investigated in our recent experimental paper published in *Nat. Nanotechnol.* **17**, 1272–1279 (2022) [39]. According to the high quality of quantum Hall plateaus (in the normal phase without charge transfer) seen at moderate magnetic field at a few or a few tens of Kelvin in these samples, we can claim that those samples, in term of cleanness, are of state-of-the-art quality.

For electrical transport measurements of the devices, we use a wire bonder to bond the device onto the sample holder (Supplementary Figure 18(a)). Samples are then loaded into a 1.5 K fridge (Oxford Tesla-Tron system), with a base temperature of 1.5K and a maximum magnetic field of 12 T. We adopt standard 4-probe low frequency (13.33 Hz) lock-in measurement method to perform electrical transport characterization of the devices, with the Standford SR830 lock-in amplifier in series with a 10 MΩ bias resistor, thus providing an AC signal at constant current of 100 nA (assume the sample resistance is way smaller than the bias resistor). Meanwhile, high precision voltage source (Keithley 2400) is used to regulate the top and bottom gate voltages. The longitudinal voltage V_{xx} is measured by the lock-in amplifier. Pictures of the set-up are shown in Supplementary Figure 19.

In addition, according to our experimental observations, we actually have two phases (see Supplementary Figure 4 in Ref.[39]) in graphene-CrOCl heterostructure, revealed in the sample's resistance measurement in the parameter space of V_{tg} and V_{bg}:

1. Phase-I is conventional graphene showing rather low Dirac peak at a few hundreds of Ohms;

2. Phase-II is the interfacial-coupling phase, where the band structure of graphene is re-configured with a mild gap opening at the charge neutral.

In fact, we focus on the Phase-II since Phase-I is the trivial conventional graphene. In the latter phase, the Wigner crystal in the substrate is not activated since the charge transfer is not onset. Given that quantum Hall plateaus have been clearly seen under sub-Tesla magnetic field at tens of Kelvin, with the Shubnikov-de Haas quantum oscillations (see Supplementary Figure 36 in Ref.[39]), the quality of sample is guaranteed. So, the measured resistivity peak has to do with gap opening in the Phase-II in CrOCl-contacted graphene. For even more experimental details, we would invite the reader to kindly refer to the Supplementary Information of *Nat. Nanotechnol.* **17**, 1272–1279 (2022) [39].

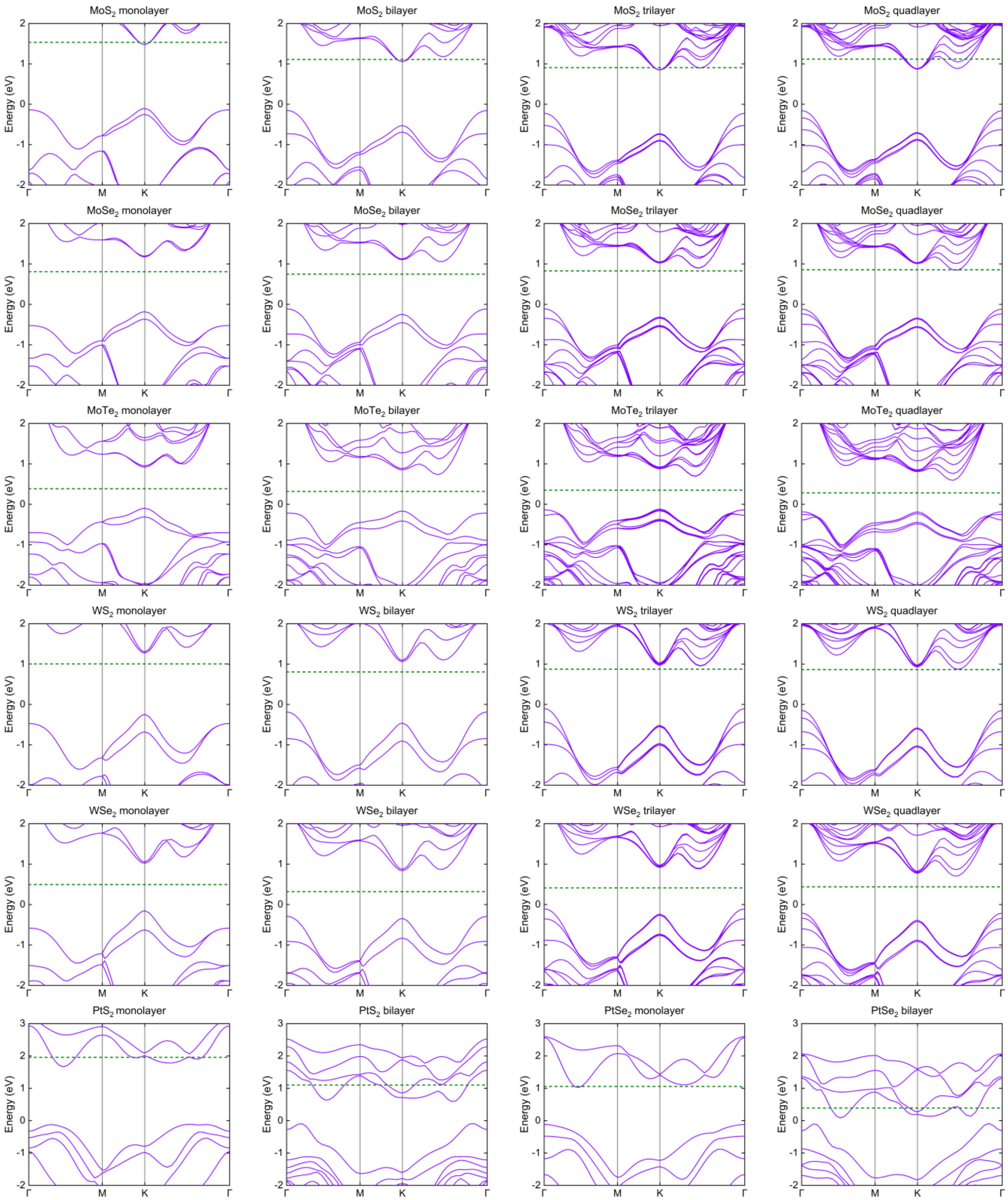

Supplementary Figure 17: The calculated energy bands of different TMD system. Here the energy positions of Dirac point in graphene monolayer are remarked with green dashed lines. The structures of MoX_2 and WX_2 systems are trigonal prismatic 2H-phase, and those of PtX_2 systems are octahedral 1T-phase, which are common structural phase. The PtS_2 and $PtSe_2$ system has strong interlayer coupling, and the trilayer will become metallic.

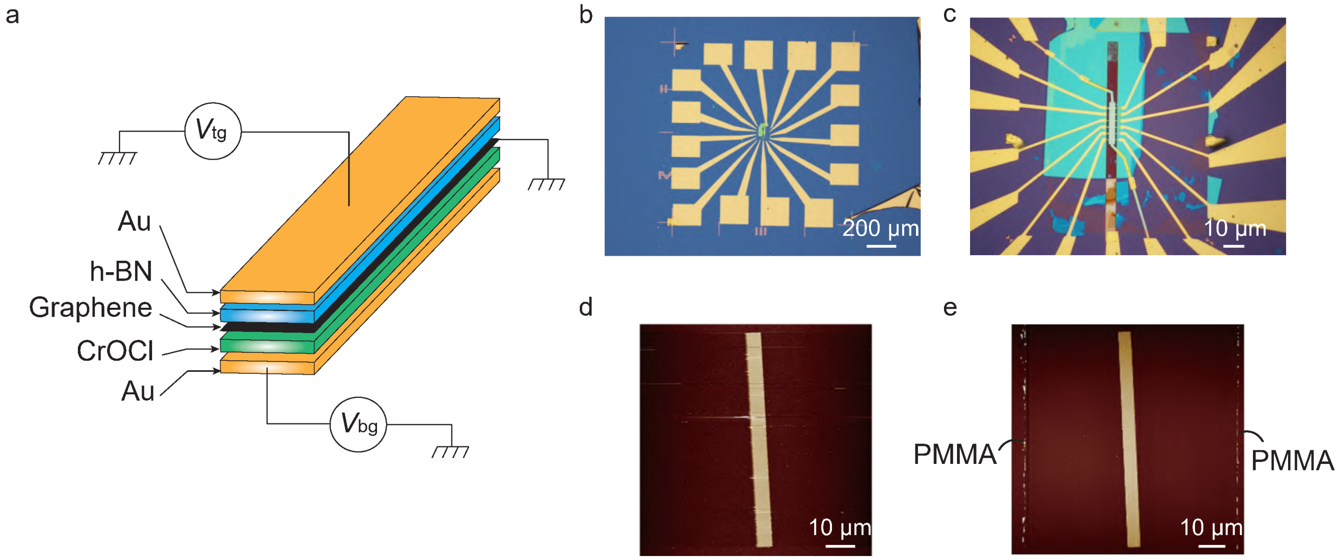

Supplementary Figure 18: The characterizations of the experimental devices. (a) The cartoon illustration of a typical h-BN/Graphene/CrOCl heterostructure device, with its optical pictures shown in (b) and (c). Scale bar in the image is 10 μm. Images of AFM scans of the Au bottom gate before (d) and after (e) AFM Contact mode cleaning are also illustrated, where the cleaned window can be highlighted by the PMMA residues accumulated during the AFM "sweeping" cleaning.

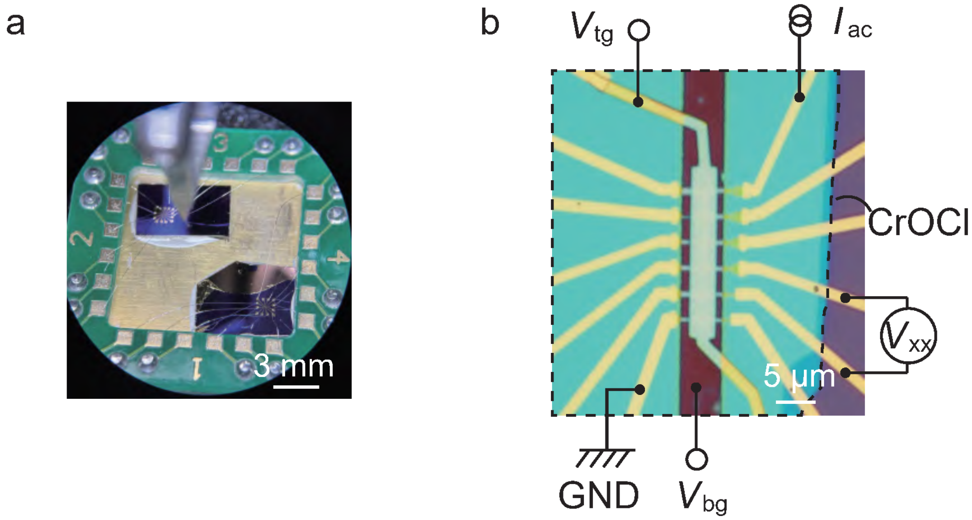

Supplementary Figure 19: Measurement configurations. (a) The image of device bonded onto the holder. (b) Diagram of the 4-probe measurement method of Hall bar. The electrode labeled "I_{ac}" is applied 100nA AC signal by SR830 with a 10 MΩ bias resistor. The longitudinal voltage V_{xx} is recorded by SR830, as well. The top gate and bottom gate electrodes are supplied voltage by Keithley 2400. Scale bar in the image is 5 μm.

Measurement of the thermal gap

The gate dependencies of channel resistances are measured at various temperatures for the extraction of thermal gaps. In Supplementary Figure 20(a), we present the detailed mapping of measured resistance in the space of displacement field $D_{\rm eff}$ and the nominal carrier density $n_{\rm tot}$. A resistivity peak which persists up to $n_{\rm tot} \lessapprox 5 \times 10^{12}\,{\rm cm}^{-2}$ is clearly seen, indicating gap opening at the charge neutrality point (CNP) of graphene. The gapped Dirac state persists up to $n_{\rm tot} \lessapprox 5 \times 10^{12}\,{\rm cm}^{-2}$ because the extra charge carriers are transferred from graphene to the

surface of CrOCl, leaving graphene at the charge neutrality. As discussed in the main text, the charges transferred to the surface of CrOCl may form a long-wavelength ordered state through the Wigner-crystallization mechanism, which imposes a superlattice Coulomb potential to graphene and promotes the gap opening at the CNP. In order to determine the gap at the CNP of graphene at different n_{tot}, we have further performed the temperature dependent conductance measurements at different n_{tot} as shown in Supplementary Figure 20(b). The temperature dependence of the conductance $\sigma_{xx}(T)$ can be very well explained in a thermal excitation picture, with $\sigma_{xx}(T) \sim e^{-\Delta/2k_BT}$ (k_B is the Boltzmann constant), from which the gap at the CNP Δ can be extracted. In Supplementary Figure 20(c) we show the experimentally measured gaps at different nominal carrier density n_{tot}. We see that the gap decreases linearly with n_{tot}, consistent with theoretical calculations.

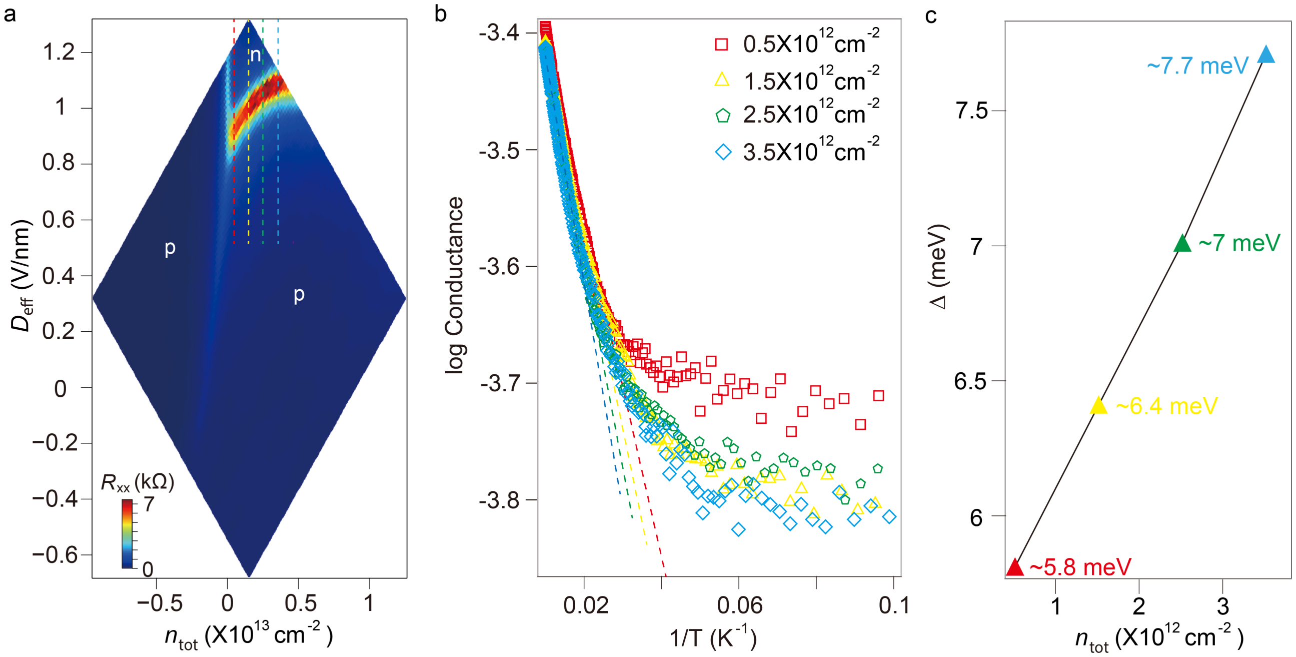

Supplementary Figure 20: Gap size at the charge neutrality point (CNP) of monolayer graphene supported by CrOCl. (a) Channel resistance R_{xx} measured in the space of D_{eff}-n_{tot} at $T = 1.5$ K and $B = 0$ T. Here, D_{eff} is defined as $D_{\text{eff}} = (C_{\text{tg}}V_{\text{tg}} - C_{\text{bg}}V_{\text{bg}})/2\epsilon_0 - D_0$ and n_{tot} is defined as $n_{\text{tot}} = (C_{\text{tg}}V_{\text{tg}} + C_{\text{bg}}V_{\text{bg}})/e - n_0$. C_{tg} and C_{bg} are the top and bottom gate capacitances per area, respectively. And V_{tg} and V_{bg} are the top and bottom gate voltages, respectively. n_0 and D_0 are residual doping and residual displacement field, respectively. A new phase driven by e-e interaction is seen, with the CNP largely bent, and clearly much more resistive than the conventional vertical CNP at the fixed $n_{tot} = 0$. This is consistent with the phase diagram reported in Ref. 39. (b) Log scale of the minimum conductance σ_{xx} at the CNP with fixed n_{tot}, i.e., $\log(1/R_{xx})$, plotted against $1/T$. The thermal excitation gap Δ can then be estimated from the linear part, using the formula $\sigma \sim e^{-\Delta/2k_BT}$, where k_B is the Boltzmann constant, T is temperature. (c) The thermal activation gap extracted in each linear fit of the curve in (b) and plotted against n_{tot}.

SUPPLEMENTARY REFERENCES

[S1] Song, Z. *et al.* All magic angles in twisted bilayer graphene are topological. *Phys. Rev. Lett.* **123**, 036401 (2019).

[S2] Ahn, J., Park, S. & Yang, B.-J. Failure of nielsen-ninomiya theorem and fragile topology in two-dimensional systems with space-time inversion symmetry: Application to twisted bilayer graphene at magic angle. *Phys. Rev. X* **9**, 021013 (2019).

[S3] Po, H. C., Zou, L., Senthil, T. & Vishwanath, A. Faithful tight-binding models and fragile topology of magic-angle bilayer graphene. *Phys. Rev. B* **99**, 195455 (2019).

[S4] Tarnopolsky, G., Kruchkov, A. J. & Vishwanath, A. Origin of magic angles in twisted bilayer graphene. *Phys. Rev. Lett.* **122**, 106405 (2019).

[S5] Liu, J., Liu, J. & Dai, X. Pseudo landau level representation of twisted bilayer graphene: Band topology and implications on the correlated insulating phase. *Phys. Rev. B* **99**, 155415 (2019).

[S6] Forsythe, C. *et al.* Band structure engineering of 2d materials using patterned dielectric superlattices. *Nature Nanotechnology* **13**, 566–571 (2018).

[S7] Ghorashi, S. A. A. *et al.* Topological and stacked flat bands in bilayer graphene with a superlattice potential. *Phys. Rev. Lett.* **130**, 196201 (2023).

[S8] Vafek, O. & Kang, J. Renormalization group study of hidden symmetry in twisted bilayer graphene with coulomb interactions. *Phys. Rev. Lett.* **125**, 257602 (2020).

[S9] Zhang, S., Dai, X. & Liu, J. Spin-polarized nematic order, quantum valley hall states, and field-tunable topological transitions in twisted multilayer graphene systems. *Phys. Rev. Lett.* **128**, 026403 (2022).

[S10] Blöchl, P. E. Projector augmented-wave method. *Phys. Rev. B* **50**, 17953–17979 (1994).

[S11] Kresse, G. & Furthmüller, J. Efficient iterative schemes for ab initio total-energy calculations using a plane-wave basis set. *Phys. Rev. B* **54**, 11169–11186 (1996).

[S12] Perdew, J. P., Burke, K. & Ernzerhof, M. Generalized gradient approximation made simple. *Phys. Rev. Lett.* **77**, 3865–3868 (1996).

[S13] Liechtenstein, A. I., Anisimov, V. I. & Zaanen, J. Density-functional theory and strong interactions: Orbital ordering in mott-hubbard insulators. *Phys. Rev. B* **52**, R5467–R5470 (1995).

[S14] Grimme, S. Semiempirical GGA-type density functional constructed with a long-range dispersion correction. *Journal of Computational Chemistry* **27**, 1787–1799 (2006).

[S15] Haastrup, S. *et al.* The computational 2d materials database: high-throughput modeling and discovery of atomically thin crystals. *2D Mater.* **5**, 042002 (2018).

[S16] Jariwala, B. *et al.* Synthesis and characterization of res$_2$ and rese$_2$ layered chalcogenide single crystals. *Chemistry of Materials* **28**, 3352–3359 (2016).

[S17] Yang, S. *et al.* Tuning the optical, magnetic, and electrical properties of rese$_2$ by nanoscale strain engineering. *Nano Letters* **15**, 1660–1666 (2015).

[S18] Arora, A. *et al.* Highly anisotropic in-plane excitons in atomically thin and bulklike 1T'-$ReSe_2$. *Nano Letters* **17**, 3202–3207 (2017).

[S19] Huang, B. *et al.* Layer-dependent ferromagnetism in a van der waals crystal down to the monolayer limit. *Nature* **546**, 270–273 (2017).

[S20] Huang, B. *et al.* Electrical control of 2D magnetism in bilayer CrI_3. *Nature Nanotechnology* **13**, 544–548 (2018).

[S21] Jiang, S., Li, L., Wang, Z., Mak, K. F. & Shan, J. Controlling magnetism in 2d CrI_3 by electrostatic doping. *Nature Nanotechnology* **13**, 549–553 (2018).

[S22] Klein, D. R. *et al.* Probing magnetism in 2D van der waals crystalline insulators via electron tunneling. *Science* **360**, 1218–1222 (2018).

[S23] Sivadas, N., Okamoto, S., Xu, X., Fennie, C. J. & Xiao, D. Stacking-dependent magnetism in bilayer CrI_3. *Nano Letters* **18**, 7658–7664 (2018).

[S24] In our DFT+U calculations, the on-site Hubbard $U = 5.48\,\mathrm{eV}$ for the Cr 3d orbitals is used in the calculations, and the non-spherical contributions from the gradient corrections are taken into consideration.

[S25] Lee, S., Colombet, P., Ouvrard, G. & Brec, R. General trends observed in the substituted thiophosphate family. synthesis and structure of silver scandium thiophosphate, $AgScP_2S_6$, and cadmium iron thiophosphate, $CdFeP_2S_6$. *Inorganic Chemistry* **27**, 1291–1294 (1988).

[S26] Seidlmayer, S., Weihrich, R. & Pfitzner, A. Synthese und kristallstruktur von $AgScP_2Se_6$. *Zeitschrift für anorganische und allgemeine Chemie* **632**, 2122–2122 (2006).

[S27] Brodersen, K., Thiele, G., Ohnsorge, H., Recke, I. & Moers, F. Die struktur des $IrBr_3$ und über die ursachen der fehlordnungserscheinungen bei den in schichtenstrukturen kristallisierenden edelmetalltrihalogeniden. *Journal of the Less Common Metals* **15**, 347–354 (1968).

[S28] Brodersen, K. Structure of β-$RuCl_3$, RuI_3, $IrBr_3$, and IrI_3. *Angewandte Chemie International Edition in English* **7**, 148–148 (1968).

[S29] Asprey, L. B., Keenan, T. K. & Kruse, F. H. Preparation and crystal data for lanthanide and actinide triiodides. *Inorganic Chemistry* **3**, 1137–1141 (1964).

[S30] Mattausch, H., Hendricks, J. B., Eger, R., Corbett, J. D. & Simon, A. Cheminform abstract: Reduced halides of yttrium with strong metal-metal bonding: Yttrium monochloride, monobromide, sesquichloride, and sesquibromide. *Chemischer Informationsdienst* **11** (1980).

[S31] Wildervanck, J. & Jellinek, F. The dichalcogenides of technetium and rhenium. *Journal of the Less Common Metals* **24**, 73–81 (1971).

[S32] Garcia, E., Corbett, J. D., Ford, J. E. & Vary, W. J. Low-temperature routes to new structures for yttrium, holmium, erbium, and thulium oxychlorides. *Inorganic Chemistry* **24**, 494–498 (1985).

[S33] Moreau, J., Kiat, J. M., Garnier, P. & Calvarin, G. Incommensurate phase in lead monoxide α-PbO below 208 k. *Phys. Rev. B* **39**, 10296–10299 (1989).

[S34] Petousis, I. *et al.* Benchmarking density functional perturbation theory to enable high-throughput screening of materials for dielectric constant and refractive index. *Phys. Rev. B* **93**, 115151 (2016).

[S35] Petousis, I. *et al.* High-throughput screening of inorganic compounds for the discovery of novel dielectric and optical materials. *Scientific Data* **4**, 160134 (2017).

[S36] Choudhary, K. *et al.* The joint automated repository for various integrated simulations (jarvis) for data-driven materials design. *npj Computational Materials* **6**, 1–13 (2020).

[S37] Drummond, N. D. & Needs, R. J. Phase diagram of the low-density two-dimensional homogeneous electron gas. *Phys. Rev. Lett.* **102**, 126402 (2009).

[S38] Ramasubramaniam, A., Naveh, D. & Towe, E. Tunable band gaps in bilayer transition-metal dichalcogenides. *Phys. Rev. B* **84**, 205325 (2011).

[S39] Wang, Y. *et al.* Quantum hall phase in graphene engineered by interfacial charge coupling. *Nature Nanotechnology* **17**, 1272–1279 (2022).
