# Supplementary material for: Synergistic correlated states and nontrivial topology in coupled graphene-insulator heterostructures
Source: Nat Commun. 2023 Sep 9;14:5550. doi: 10.1038/s41467-023-41293-8 (PMC10492827; doi:10.1038/s41467-023-41293-8)
Supplement: Supplementary file 3 — Description of Additional Supplementary Files [file 41467_2023_41293_MOESM3_ESM.pdf]

Description of Additional Supplementary Files

File Name: Supplementary Movie 1:

Description: Evolution of band structure with the increase of anisotropy r, for superlattice constant Ls=50Å

File Name: Supplementary Movie 2:

Description: Evolution of Berry curvature with the increase of anisotropy r, for superlattice constant Ls=50Å

File Name: Supplementary Movie 3:

Description: Evolution of band structure with the increase of anisotropy r, for superlattice constant Ls=200Å

File Name: Supplementary Movie 4:

Description: Evolution of Berry curvature with the increase of anisotropy r, for superlattice constant Ls=200Å

File Name: Supplementary Movie 5:

Description: Evolution of band structure with the increase of anisotropy r, for superlattice constant Ls=600Å

File Name: Supplementary Movie 6:

Description: Evolution of Berry curvature with the increase of anisotropy r, for superlattice constant Ls=600Å
